# CRE-Ter enhances murine bone differentiation, improves muscle cell atrophy, and increases irisin expression

**DOI:** 10.1371/journal.pone.0338571

**Published:** 2025-12-04

**Authors:** Sompot Jantarawong, Wipapan Khimmaktong, Pharkphoom Panichayupakaranant, Yutthana Pengjam

**Affiliations:** 1 Faculty of Medical Technology, Prince of Songkla University, Hat Yai, Songkhla, Thailand; 2 Division of Health and Applied Sciences, Faculty of Science, Prince of Songkla University, Hat Yai, Songkhla, Thailand; 3 Department of Pharmacognosy and Pharmaceutical Botany, Faculty of Pharmaceutical Sciences, Prince of Songkla University, Hat Yai, Songkhla, Thailand; 4 Phytomedicine and Pharmaceutical Biotechnology Excellence Center, Faculty of Pharmaceutical Sciences, Prince of Songkla University, Hat Yai, Songkhla, Thailand; University of Sahiwal, PAKISTAN

## Abstract

Ternary complex of curcuminoid-rich extract (CRE-Ter) is a developed water-soluble *Curcuma longa* extract containing 14% *w/w* curcuminoids, hydroxypropyl-β-cyclodextrin, and polyvinylpyrrolidone K30. This study aimed to investigate the biomolecular effects of CRE-Ter on differentiation of bone cells (murine MC3T3-E1 preosteoblasts), muscle cells (murine dexamethasone-treated C2C12 myotubes) atrophy and irisin expression. In MC3T3-E1 preosteoblasts, CRE-Ter treatment increased alkaline phosphatase activity, calcium deposition, and expression of Bmp-2, Runx2, and collagen 1a significantly and dose-dependently. 5, 10, and 20 µg/mL CRE-Ter upregulated β-catenin expression significantly. CRE-Ter improved the atrophy of dexamethasone-treated C2C12 myotubes. CRE-Ter decreased proinflammatory cytokine (TNF-α and IL-6) expression but increased FNDC5 and irisin expression and nitric oxide production in dexamethasone-treated C2C12 myotubes significantly and dose-dependently. Dexamethasone promoted β-catenin and total p38 expression in C2C12 myotubes. CRE-Ter at 2.5–20 µg/mL reversed the increase in β-catenin expression, whereas 2.5 µg/mL reversed total p38 expression. Crosstalk experiments further revealed that conditioned medium from C2C12 myotubes enhanced osteocalcin expression in MC3T3-E1 osteoblasts. Molecular docking simulations using CB-Dock2 showed strong interactions between each curcuminoid molecule and irisin. Therefore, CRE-Ter may stimulate osteoblast differentiation, ameliorate myotube atrophy, and increase irisin expression, indicating its therapeutic potential in osteoporosis, sarcopenia, and osteosarcopenia.

## Introduction

Osteoporosis and sarcopenia are musculoskeletal disorders that have emerged as critical health concerns worldwide, especially among the older population [1]. Osteoporosis is characterized by low bone density, deteriorated bone structure, increased bone fragility, and a high risk of fracture [1–4]. Sarcopenia is the progressive and widespread loss of skeletal muscle mass that leads to a high risk of falls and fractures, poor mobility, and high mortality [5–11]. Osteosarcopenia is the coexistence of osteoporosis and sarcopenia. Some clinical trials have reported that either osteoporosis or sarcopenia could develop into osteosarcopenia, resulting in increased risks of fragility and disability [12,13]. Osteosarcopenia may be attributed to the coupling biomolecular mechanism between bone and muscle [13]. Consequently, investigating such crosstalk is essential for improving treatment modalities in osteoporosis, sarcopenia, and osteosarcopenia.

In bone development, osteoblasts (bone-forming cells) and osteoclasts (bone-resorbing cells) have a pivotal role in maintaining bone homeostasis. Excessive osteoclast activity and limited osteoblast activity lead to bone resorption and bone fragility [14]. Osteoblasts are differentiated from preosteoblasts [14]. They secrete calcium and hydroxyapatite, which stimulate bone mineralization, as well as extracellular matrix proteins, such as alkaline phosphatase (ALP), collagen 1a, and osteocalcin [14]. The production of these markers follows a temporal pattern: collagen synthesis predominates during the proliferative phase, ALP expression reaches its peak during matrix maturation, and osteocalcin secretion becomes most prominent during mineralization [15]. Canonical Wnt and bone morphogenetic protein (BMP) signaling pathways are key regulators in the promotion of osteoblast differentiation. In the canonical Wnt signaling pathway, the Wnt ligand interacts with frizzled and low-density lipoprotein receptor-related protein 5 or 6, upregulating β-catenin and Runt-related transcription factor 2 (Runx2) expression. In BMP signaling, Bmp-2 binds to Bmp-2 receptor on the cell surface, activating the expression of Runx2 [14].

Among bone-derived factors, osteocalcin plays a central role in bone–muscle crosstalk. Osteocalcin is the most abundant non-collagenous protein in bone matrix and its expression is transcriptionally regulated by Runx2, the master regulator of osteoblast differentiation. Beyond osteoblasts, circulating osteogenic precursor cells also express osteocalcin together with markers such as ALP and type I collagen, highlighting its broader role in bone remodeling mineralization [15]. Once released into circulation, osteocalcin acts as an endocrine hormone that enhances muscle protein synthesis, preserves muscle mass, and improves metabolic adaptation during exercise mineralization [15]. In vivo studies showed that osteocalcin-deficient mice exhibit reduced muscle mass, whereas exogenous osteocalcin supplementation in mice increases protein synthesis in myotubes and muscle mass and prevents age-related muscle loss mineralization [15]. Mechanical loading and physical training further stimulate osteocalcin production, which contributes to enhanced musculoskeletal performance and bone strength mineralization [15]. Importantly, osteocalcin signaling in myofibers promotes glucose and fatty acid utilization during exercise and triggers the release of interleukin-6 (IL-6). IL-6 subsequently activates RANKL expression in osteoblasts, leading to further osteocalcin release from bone and establishing an osteocalcin–IL-6–RANKL endocrine axis that sustains muscle function mineralization [15]. These findings position osteocalcin as both a structural and endocrine mediator that couples bone metabolism to muscle physiology, underscoring its therapeutic relevance in osteosarcopenia.

Inflammation is a major factor that leads to muscle atrophy and the deterioration of muscle regeneration in patients with sarcopenia [16]. During muscle injury, numerous growth factors and cytokines activate satellite cells that proliferate to form myoblasts. Myoblasts differentiate into myocytes. The fusion of myocytes leads to the formation of myotubes. The myotubes mature into new myofibers to modulate muscle regeneration [17–20]. Inflammatory signaling pathways, such as the canonical mitogen-activated protein kinase (MAPK) and canonical Wnt signaling pathways, lead to sarcopenia [21]. In the canonical MAPK signaling pathway, cytokines are upstream stimuli that trigger the phosphorylation of p38 kinases [22]. Cytokines can give rise to the transcription of proinflammatory cytokines, such as TNF-α and IL-6, in skeletal muscle cells through the canonical MAPK signaling pathway [23]. Many *in vitro* investigations have supported the hypothesis that canonical Wnt ligands stimulate myoblast differentiation [24]. The upregulation of nitric oxide (NO) signaling can improve the regeneration of injured skeletal muscle by stimulating satellite cell activation, proliferation, and myogenesis, as well as enhancing macrophage activity and early phase repair [25].

Irisin is a myokine derived from the cleavage of fibronectin type III domain-containing protein 5 (FNDC5) [26]. Many studies have suggested that irisin may be utilized in the screening and treatment of osteoporosis, sarcopenia, and osteosarcopenia [27–30]. Irisin can promote bone formation through the canonical MAPK and canonical Wnt signaling pathways, resulting in the increased expression of Runx2 and ALP [27–29]. Irisin promoted the formation of skeletal muscle cells, increased skeletal muscle mass and strength, inhibited the atrophy of dexamethasone (Dex)-induced C2C12 myotubes, and suppressed muscle loss and atrophy [27,28,30]. Despite its prospects in the clinic, irisin has a short half-life. Exogenous irisin supplementation may increase irisin levels; however, exogenous irisin may exhibit side effects [31] and the response to irisin in rodents and humans can vary [29,31,32]. Hence, adopting alternative approaches to treat osteoporosis, sarcopenia, and osteosarcopenia by modulating the endogenous irisin generated in muscle cells may overcome these challenges.

**Fig 1:**
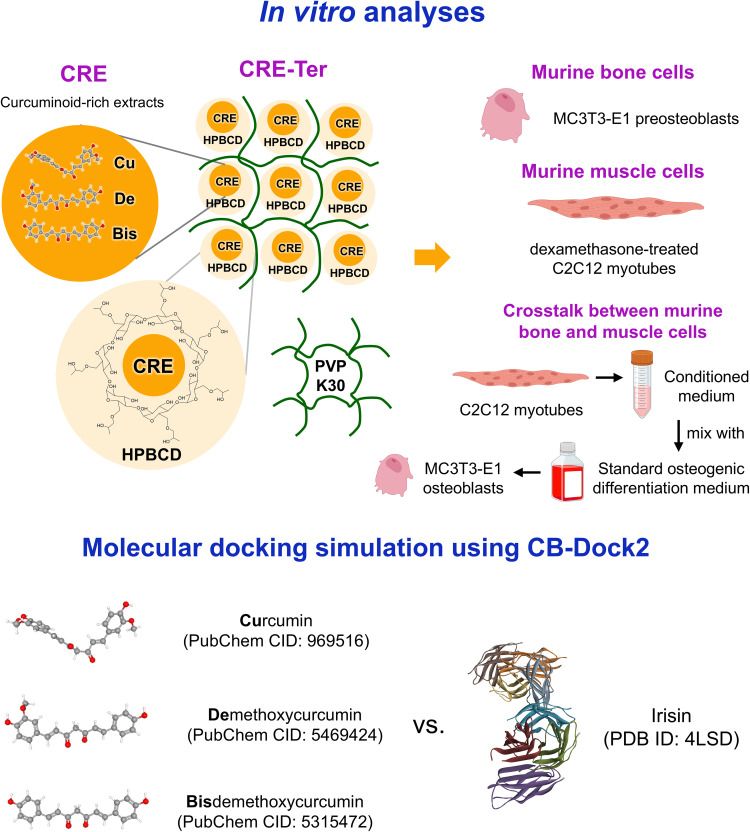
Schematic showing the concept of this study. The dried powder of *Curcuma longa* rhizomes was used to prepare the curcuminoid-rich extract (CRE) containing 88% *w/w* total curcuminoids, including curcumin (Cu), demethoxycurcumin (De), and bisdemethoxycurcumin (Bis). CRE was incorporated with 9% *w/w* polyvinylpyrrolidone K30 (PVP K30) and hydroxypropyl-β-cyclodextrin (HPBCD) at a molar ratio of 1:1 to produce the ternary complex of CRE (CRE-Ter), containing 14% *w/w* curcuminoids. For *in vitro* analyses, murine bone cells (MC3T3-E1 preosteoblasts) and muscle cells (dexamethasone-treated C2C12 myotubes) were treated with CRE-Ter. For molecular docking simulation, the CB-Dock2 website was employed to examine the interactions between each curcuminoid molecule and irisin.

Turmeric (*Curcuma longa* L.) is a rhizomatous and perennial plant of the *Zingiberaceae* or ginger family [33,34]. It has anti-inflammatory and antioxidant effects and has consequently been used in herbalism [33]. Curcuminoids, the major active phenolic compounds in turmeric, contain ~80% curcumin (Cu), demethoxycurcumin (De), and bisdemethoxycurcumin (Bis) [35]. Many clinical trials have shown that curcuminoids are safe, have no serious side effects, and have high tolerability and therapeutic efficacy [36]. Preclinical and clinical studies have shown that curcumin can prevent osteoporosis by stimulating osteoblast differentiation [37]. Wei et al. showed that Bis can mitigate osteoporosis by promoting osteoblast differentiation [38]. Many pharmacological studies have suggested that curcumin is a promising medicine for sarcopenia, because it can increase satellite cell development; increase muscle mass; and inhibit inflammation, muscle atrophy, and muscle damage [39,40]. Huang et al. showed that a combination of Cu, De, and Bis had synergistic effects on HOS osteosarcoma cells, compared with when one or two curcuminoids were used [41]. Notably, curcumin increased the expression of circulating irisin [42,43]. Phytotherapeutic potential notwithstanding, curcumin is lipophilic and has low polarity, chemical stability, and dissolution, leading to limited oral bioavailability, poor absorption, and rapid metabolism [44]. To ameliorate these pharmacokinetic properties, we developed a ternary complex of curcuminoid-rich extract (CRE) called CRE-Ter. Our studies have shown that CRE-Ter has a higher solubility than Cu, De, and Bis [45]. The biomolecular regulation of CRE-Ter in osteoblast differentiation, myotube atrophy, and irisin expression remains unknown. To our knowledge, no prior bioinformatics studies have evaluated whether each curcuminoid molecule has a strong binding affinity to irisin. In this study, we evaluated these biomolecular mechanisms *in vitro* using cultured murine MC3T3-E1 preosteoblasts and C2C12 myoblasts. We further established a crosstalk model by culturing MC3C3-E1 osteoblasts with conditioned medium derived from C2C12 myotubes to examine how muscle-derived factors influence osteoblast differentiation and how CRE-Ter modulates this mechanism. Additionally, to support the experimental findings, we analyzed the interactions between each curcuminoid molecule and irisin using molecular docking simulation. The conceptual framework of this study is shown in Fig 1.

## Materials and methods

### Cells, reagents, and instruments

Sources of cells, reagents, and instruments are detailed in Table S1 in S2 File.

### CRE-Ter preparation

CRE-Ter, which contains 14% *w/w* curcuminoids, was prepared as described [45,46]. The dried powder of *C. longa* rhizomes was suspended in ethanol. A microwave-assisted extraction was performed at 900 W, 70°C–75°C, and three irradiation cycles (one cycle: 3 min power-on and 30 s power-off). The resulting extract was filtered and fractionated through a Diaion HP-20 column that was eluted with the incorporation of 55% and 60% *v*/*v* ethanol. This led to the production of CRE, containing 89.54% *w/w* total curcuminoids (72.81% ± 0.83% *w/w* Cu, 12.49% ± 0.57% *w/w* De, and 4.24% ± 0.16% *w/w* Bis). The curcuminoid content of CRE was measured using HPLC [46].

To obtain CRE-Ter, CRE was mixed with hydroxypropyl-β-cyclodextrin at a molar ratio of 1:1 using solvent evaporation to produce an inclusion complex, and dispersed in 9% *w/w* polyvinylpyrrolidone K30 to produce a solid dispersion. Then, curcuminoid was evaporated under reduced pressure conditions. According to HPLC analysis, the resulting CRE-Ter contains 14% *w/w* curcuminoids [45].

### Cell culture and cell viability analysis

Murine (*Mus musculus*) MC3T3-E1 preosteoblasts and C2C12 myoblasts were cultured in α-MEM and DMEM culture media, respectively. Before use, 10% fetal bovine serum and antibiotic–antimycotic solution were added to all culture media. All cell lines were incubated in a humidified chamber (5% CO_2_ and 37°C). Confluent MC3T3-E1 preosteoblasts were cultured on 6-well plates containing standard osteogenic differentiation medium (α-MEM supplemented with 50 mg/mL ascorbic acid, 5 mM β-glycerophosphate, and 100 nM dexamethasone to induce osteoblast differentiation, as stated [47]. Semiconfluent C2C12 myoblasts were cultured on 6-well plates containing DMEM medium with 2% horse serum and antibiotic–antimycotic solution for 1 day to induce differentiation into C2C12 myotubes. Then, C2C12 myotubes were treated with 1 μM Dex for 24 h to mimic muscle atrophy [48]. MC3T3-E1 preosteoblasts and Dex-treated C2C12 myotubes were treated with CRE-Ter for further experiments.

To investigate the crosstalk between osteoblasts and myotubes, semiconfluent C2C12 myoblasts were cultured in 6-well plates with DMEM supplemented with 2% horse serum and antibiotic–antimycotic solution and incubated for 1 day. Then, C2C12 myotubes were treated with 1 μM Dex for 24 h, after which the conditioned medium was collected. MC3T3-E1 preosteoblasts were then cultured for 14 days under two conditions: (1) standard osteogenic differentiation medium and (2) standard osteogenic differentiation medium supplemented with 10% *v*/*v* conditioned DMEM from C2C12 myotubes. For each condition, cells were further treated with CRE-Ter at 5, 10, or 20 μg/mL.

Cell viability analysis was performed as stated [49,50]. 3 × 10^3^ cells/mL of MC3T3-E1 preosteoblasts and Dex-treated C2C12 myotubes were seeded in 96-well plates for 1 day. Then, 10 µL of 1 mg/mL MTT solution was added to each sample well, and the cells were incubated in a humidified chamber for 4 h. After the incubation period, the supernatant was discarded and 100 µL of dimethyl sulfoxide was added to each sample well. The 96-well plates were shaken for 10 min, and absorbance was measured using a microplate spectrophotometer at 570 nm.

### Measurement of ALP activity and ALP staining

MC3T3-E1 preosteoblasts were treated with CRE-Ter for 10 days, washed twice with cold phosphate-buffered saline, and lysed in cell lysis buffer. An ALP activity kit was used to measure ALP activity. Cell lysates were incubated in ALP substrate buffer (100 mmol/L Tris-HCl [pH 8.5], 2 mmol/L MgCl_2_, and 6.6 mmol/L 4-nitrophenyl phosphate) for 30 min. Absorbance was measured at 405 nm using a microplate reader. The absorbance value was used to calculate ALP activity. An ALP staining kit was used for ALP staining. MC3T3-E1 preosteoblasts were treated with 20 μg/mL CRE-Ter for 10 days in a humidified chamber, washed twice with phosphate-buffered saline, fixed with 10% neutral buffered formalin for 20 min at room temperature, washed with deionized water, incubated with the chromogenic substrate at 37ºC for 20 min, dehydrated at room temperature for 1 h, and imaged using a brightfield microscope integrated with a digital camera (photographed at 10 × magnification with the objective lens).

### Alizarin red S staining

Alizarin red S staining was performed to investigate calcium deposition in MC3T3-E1 preosteoblasts. MC3T3-E1 preosteoblasts were treated with 20 μg/mL CRE-Ter for 21 days, fixed in 4% paraformaldehyde, and incubated in 1% Alizarin red S solution for 5 min at room temperature. Intracellular Ca^2+^ deposition was observed using a brightfield microscope. Absorbance was measured at 550 nm using a spectrophotometer.

### Morphology analysis of C2C12 myotubes

C2C12 myotubes were visualized and photographed at 10 × magnification using an EVOS M5000 inverted microscope. Myotube size was measured using ImageJ software (https://imagej.net/ij/). Two orthogonal lines were drawn through the center of each image, and myotube diameter was measured at the intersection points of the said lines. An independent observer, blinded to culture conditions, performed the morphometric analysis.

### Measurement of NO production

We used a colorimetric assay to measure the concentration of nitrite, a soluble oxidation product of NO, released from the cells. Dex- and CRE-Ter-treated C2C12 myotubes (5 × 10^5^ cells/mL) were seeded on a 96-well plate overnight. Dimethyl sulfoxide-treated C2C12 myotubes (5 × 10^5^ cells/mL) were used as the negative control. Then, 50 µL of Griess reagent (1% sulfanilamide and 0.1% *N*-(1-naphthyl)-ethylenediamine dihydrochloride in 5% phosphoric acid) was mixed with 50 µL of the cell supernatant. Absorbance was measured at 550 nm excitation and 580 nm emission wavelengths using a microplate reader.

### Detection of cytokines using ELISA

The detection of TNF-α and IL-6 was performed using a modified protocol based on Jantarawong et al. [49]. C2C12 myotubes were cultured on 6-well plates (1 × 10^5^ cells/well), incubated overnight, and treated with CRE-Ter for 1 h and then with Dex for 6 h. Cells treated with normal saline were used as the negative control. The cell supernatant was collected by centrifugation. TNF-α and IL-6 concentrations in each sample were measured using mouse TNF-α and IL-6 ELISA kits, respectively.

### Real-time RT-PCR

Total RNA from murine MC3T3-E1 preosteoblasts and C2C12 myotubes (5 × 10^3^ cells/µL) was isolated using RNeasy Mini Kit. cDNA was synthesized using ReverTra Ace qPCR Kit. Fast-Start SYBR Green Master Mix was used to perform real-time RT-PCR and detect the expression of Bmp-2, Runx2, collagen 1a, TNF-α, IL-6, FNDC5, irisin, and osteocalcin genes. The relative expression of the target genes to GAPDH was calculated using a Molecular Imager integrated with a chemiluminescent image analyzer. The primers used as reported [51–54] are listed in Table S2 in S2 File.

### Western blotting

Proteins were extracted from the cell lysates of murine MC3T3-E1 preosteoblasts and C2C12 myotubes using RIPA buffer. The lysates were centrifuged at 10000 rpm for 10 min at 4°C. The supernatant containing the proteins was collected. SDS-PAGE was employed to separate the proteins on 12% acrylamide gel (at 100 V for 90 min). The proteins were transferred to polyvinyl difluoride membranes (100 V, 60 min). The membranes were blocked with 5% nonfat milk in TBS-Tween at room temperature for 4 h and incubated at 4°C overnight with anti-β-catenin, anti-total p38, and anti-β-actin diluted 1:1000 in blocking buffer. Each specific primary antibody was incubated on entirely separate blots. The membranes were washed with TBS-Tween three times for 10 min each, incubated with horseradish peroxidase-conjugated secondary antibodies (diluted 1:10000 in blocking buffer) at room temperature for 2 h, and washed with TBS-Tween three times for 10 min each. All antibodies were tested on separate membranes. The protein bands were detected using an enhanced chemiluminescence substrate and visualized using the Chemiluminescence Vilber (Fusion Solo S) digital imaging system.

### Ethics statement

The research described in this manuscript did not involve human participants, human specimens, or animal experiments. Only established murine cell lines and computational analyses were used; thus, no ethical approval was necessary.

### Statistical analysis

The MTT assay of MC3T3-E1 preosteoblasts and C2C12 myotubes was conducted independently in eight replicates. Measurement of ALP activity and Alizarin red S activity in MC3T3-E1 preosteoblasts, and measurement of NO production in C2C12 myotubes were performed independently in six replicates. The other *in vitro* experiments were performed independently in triplicate. Descriptive statistics and Cohen’s *d* were calculated using Microsoft Excel (Version 2410). Numeric data are expressed as mean ± standard deviation. Cohen’s *d* was calculated by dividing the mean difference between treatment and control groups by the pooled standard deviation. Effect sizes were interpreted as small (|Cohen’s *d*| = 0.2), moderate (|Cohen’s *d*| = 0.5), and large (|Cohen’s *d*| = 0.8). Data normality was assessed using the Shapiro-Wilk test (https://www.statskingdom.com/shapiro-wilk-test-calculator.html). Statistical analyses of the significance of differences among groups were calculated using one-way analysis of variance in Microsoft Excel (Version 2410), followed by *post hoc* one-tailed Student’s *t*-test [Microsoft Excel (Version 2410)] and Tukey’s HSD test (https://www.statskingdom.com/180Anova1way.html). Statistical significance was defined as a *p*-value of <0.05. The *p*-values calculated in the one-tailed Student’s *t*-*t*est are reported in the Results section.

### Molecular docking simulation

The 3D chemical structure of each curcuminoid molecule was retrieved from the PubChem database [55] in SDF format (accessed on December 20, 2022). The PubChem compound identifiers of Cu, De, and Bis are 969516, 5469424, and 5315472, respectively. The 3D structure of irisin (Protein Data Bank (PDB) ID: 4LSD) was retrieved from RCSB PDB (https://www.rcsb.org/) [56] in PDB format (accessed on January 17, 2024). We used CB-Dock2 (https://cadd.labshare.cn/cb-dock2/php/index.php) [57] for molecular docking simulations between each curcuminoid molecule and irisin. The 3D structure of each ligand (Cu, De, or Bis) and irisin was submitted to CB-Dock2 to prepare the structures of irisin and ligand and identify binding pockets of irisin. The five largest binding pockets of irisin were detected and used for structure-based ligand–protein docking simulations using the AutoDock Vina algorithm (version 1.1.2) in CB-Dock2. The output with the most negative Vina score, indicating the highest binding affinity between each curcuminoid molecule and irisin, was selected for analysis. BIOVIA discovery studio visualizer (version 21.1.0.20298; https://discover.3ds.com/discovery-studio-visualizer-download/) was used to visualize the curcuminoid–irisin interactions.

## Results

### Effects of CRE-Ter in MC3T3-E1 preosteoblasts

**Fig 2:**
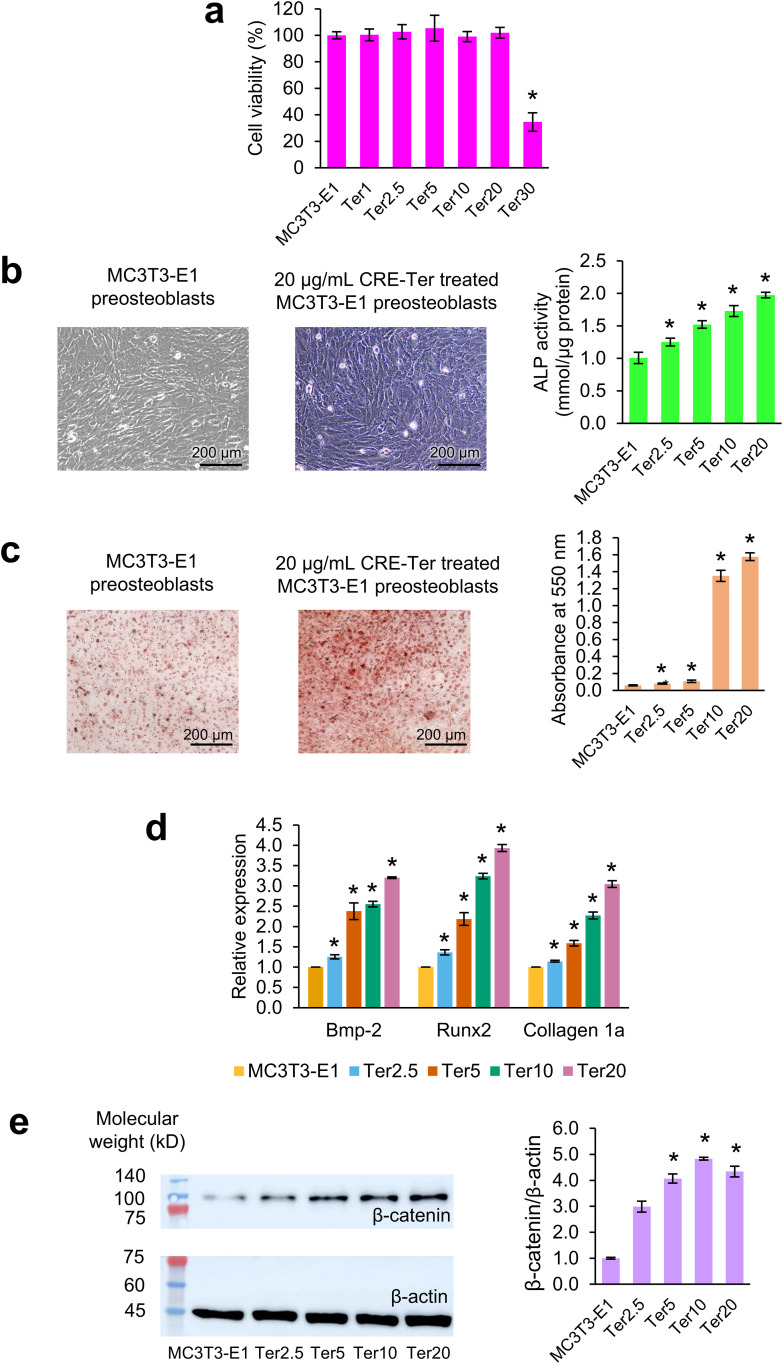
The regulation of CRE-Ter in the differentiation of MC3T3-E1 preosteoblasts. **(a)** Percent cell viability. **(b)** Brightfield microscopy images of the cells after alkaline phosphatase (ALP) staining and ALP activity. **(c)** Brightfield microscopy images of the cells after Alizarin red S staining and absorbance at 550 nm. **(d)** Relative expression of Bmp-2, Runx2, and collagen 1a normalized to GAPDH. **(e)** Western blot images and relative expression of β-catenin normalized to β-actin. MC3T3-E1 preosteoblasts were treated with 1, 2.5, 5, 10, and 20 µg/mL of CRE-Ter (Ter1, Ter2.5, Ter5, Ter10, and Ter20, respectively). The statistical significance of the difference between CRE-Ter treated and untreated cells is denoted as ^*^p < 0.05. *n* = 8 for the MTT assay, *n* = 6 for ALP activity and Alizarin red S activity, and *n* = 3 for others.

The difference of cell viability between CRE-Ter-treated and untreated MC3T3-E1 preosteoblasts was not statistically significant and the effect size was small to moderate (*p*-values: Ter1 = 0.4140, Ter2.5 = 0.0932, Ter5 = 0.0864, Ter10 = 0.3038, and Ter20 = 0.1257) (Fig 2a and Tables S3–S5 in S2 File). However, treatment of 30 μg/mL CRE-Ter decreased cell viability of MC3T3-E1 preosteoblasts significantly and the effect size was large (*p*-value = 5.6034 × 10^−9^). CRE-Ter treatment increased ALP activity in MC3T3-E1 preosteoblasts significantly and dose-dependently with a large effect size (*p*-values: Ter2.5 = 0.0015, Ter5 = 9.6129 × 10^−7^, Ter10 = 2.2796 × 10^−5^, and Ter20 = 8.2030 × 10^−7^) (Fig 2b and Tables S6–S9 in S2 File). CRE-Ter increased intracellular calcium deposition statistically significantly and dose-dependently with a large effect size (*p*-values: Ter2.5 = 0.0013, Ter5 = 4.4699 × 10^−5^, Ter10 = 3.7245 × 10^−8^, and Ter20 = 1.9001 × 10^−9^) (Fig 2c and Tables S10–S13 in S2 File). Thus, CRE-Ter stimulates differentiation and calcium deposition in MC3T3-E1 preosteoblasts. CRE-Ter treatment of MC3T3-E1 preosteoblasts increased the expression of osteoblast marker genes (Bmp-2, Runx2, and collagen 1a) statistically significantly and dose-dependently with large effect sizes (*p*-values of Bmp-2: Ter2.5 = 0.0069, Ter5 = 0.0037, Ter10 = 3.2222 × 10^−4^, and Ter20 = 1.3551 × 10^−5^; *p*-values of Runx2: Ter2.5 = 0.0049, Ter5 = 0.0028, Ter10 = 1.5117 × 10^−4^, and Ter20 = 1.3836 × 10^−4^; and *p*-values of collagen 1a: Ter2.5 = 0.0037, Ter5 = 0.0022, Ter10 = 7.5735 × 10^−4^, and Ter20 = 2.8291 × 10^−4^) (Fig 2d and Tables S14–S25 in S2 File). Western blot analysis showed a significant increase in β-catenin expression in 5, 10, and 20 μg/mL CRE-Ter-treated MC3T3-E1 preosteoblasts (*p*-values for Ter5 = 0.0380, Ter10 = 0.0031, and Ter20 = 0.0463). The effect size of CRE-Ter treatment on β-catenin expression was large. However, this increase was not statistically significant after treatment with 2.5 μg/mL CRE-Ter (*p*-value = 0.1071) (Fig 2e, Figs S1–S6 in S1 File, and Tables S26–S28 in S2 File).

### Effects of CRE-Ter in C2C12 myotubes

**Fig 3:**
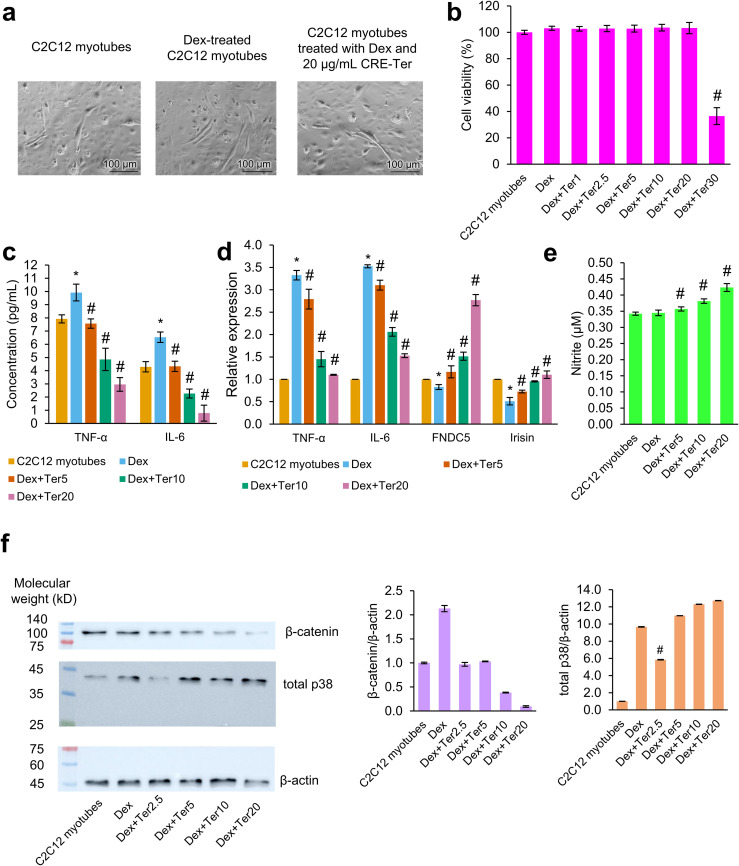
The regulation of CRE-Ter in dexamethasone (Dex)-treated C2C12 myotubes. **(a)** Microscopy images of C2C12 myotubes. **(b)** Percent cell viability. **(c)** The concentrations of TNF-α and IL-6 in C2C12 myotubes. **(d)** Relative expression of TNF-α, IL-6, FNDC5, and irisin normalized to GAPDH. **(e)** Concentration of nitrite, an indirect indicator of nitric oxide production, in C2C12 myotubes. **(f)** Western blot images and relative expression of β-catenin and total p38 normalized to β-actin. C2C12 myotubes treated with Dex are denoted as Dex in the legends and *x*-labels of bar charts and Western blot images. Dex-treated C2C12 myotubes were further treated with 1, 2.5, 5, 10, and 20 µg/mL of CRE-Ter (denoted as Dex + Ter1, Dex + Ter2.5, Dex + Ter5, Dex + Ter10, and Dex + Ter20, respectively). Statistical significance of the difference between Dex-treated and untreated C2C12 myotubes is denoted as ^*^p < 0.05. Statistical significance of the difference between CRE-Ter treated and untreated Dex-treated C2C12 myotubes is denoted as ^#^*p* < 0.05. *n* = 8 for MTT assay, *n* = 6 for the measurement of nitric oxide production, and *n* = 3 for others.

Dex induced atrophy in C2C12 myotubes (decreased diameter) that was rescued by 20 μg/mL CRE-Ter (Fig 3a). The result of the MTT assay (Fig 3b and Tables S29–S31 in S2 File) showed that Dex-treated C2C12 myotubes had a significantly higher proportion of cell viability than C2C12 myotubes not treated with Dex and CRE-Ter (*p*-value = 3.2420 × 10^−4^). There was no cytotoxicity in C2C12 myotubes treated with Dex and 1–20 μg/mL CRE-Ter, confirmed by the absence of statistically significant differences between C2C12 myotubes treated with Dex and those treated with Dex and 1–20 μg/mL CRE-Ter (*p*-values: Dex + Ter1 = 0.3218, Dex + Ter2.5 = 0.4030, Dex + Ter5 = 0.4014, Dex + Ter10 = 0.3112, and Dex + Ter20 = 0.4665). However, 30 μg/mL CRE-Ter suppressed the cell viability of Dex-treated C2C12 myotubes significantly (*p*-value = 1.2792 × 10^−8^). The effect sizes of Dex and CRE-Ter on cytotoxicity were large. The findings of ELISA (Fig 3c and Tables S32–S39 in S2 File) demonstrated that Dex significantly increased TNF-α and IL-6 concentrations in C2C12 myotubes (*p*-value of TNF-α = 0.0172 and *p*-value of IL-6 = 0.0145). By contrast, CRE-Ter substantially reduced TNF-α and IL-6 concentrations in Dex-treated C2C12 myotubes dose-dependently (*p*-values of TNF-α: Dex + Ter5 = 0.0270, Dex + Ter10 = 0.0096, and Dex + Ter20 = 0.0018 and *p*-values of IL-6: Dex + Ter5 = 0.0165, Dex + Ter10 = 0.0039, and Dex + Ter20 = 0.0043). The effect sizes of Dex and CRE-Ter on TNF-α and IL-6 concentrations were large, but Dex and 5 μg/mL of CRE-Ter had a very small effect on IL-6 concentration. The results of real-time RT-PCR analysis were consistent, as demonstrated by dose-dependent and significant changes and large effect sizes in all treatment conditions (*p*-values of TNF-α: Dex = 3.4608 × 10^−4^, Dex + Ter5 = 0.0388, Dex + Ter10 = 0.0019, and Dex + Ter20 = 3.0932 × 10^−4^ and *p*-values of IL-6: Dex = 2.8704 × 10^−5^, Dex + Ter5 = 0.0138, Dex + Ter10 = 9.3420 × 10^−4^, and Dex + Ter20 = 2.8976 × 10^−5^) (Fig 3d and Tables S40–S47 in S2 File). Dex decreased FNDC5 and irisin expression in C2C12 myotubes significantly with a large effect size (*p*-values: FNDC5 = 0.0162 and irisin = 0.0048) (Fig 3d and Tables S48–S55 in S2 File). CRE-Ter treatment significantly and dose-dependently increased FNDC5 and irisin expression in Dex-treated C2C12 myotubes and its effect size was large (*p*-values: FNDC5: Dex + Ter5 = 0.0455, Dex + Ter10 = 0.0072, and Dex + Ter20 = 3.2556 × 10^−4^; irisin: Dex + Ter5 = 0.0403, Dex + Ter10 = 0.0061, and Dex + Ter20 = 1.9130 × 10^−4^). We investigated NO production in C2C12 myotubes treated with Dex and CRE-Ter using nitrite concentration as an indicator. The difference in NO production between Dex-treated C2C12 myotubes and C2C12 myotubes not treated with Dex and CRE-Ter was not statistically significant and Dex’s effect size was small to moderate (*p*-value = 0.2429) (Fig 3e and Tables S56–S59 in S2 File). Nevertheless, CRE-Ter increased NO production in Dex-treated C2C12 myotubes significantly and dose-dependently with large effect sizes (*p*-values: Dex + Ter5 = 1.3088 × 10^−7^, Dex + Ter10 = 6.9824 × 10^−9^, and Dex + Ter20 = 6.2289 × 10^−8^). Western blot analysis showed that Dex treatment considerably increased the expression of β-catenin and total p38 proteins in C2C12 myotubes (*p*-value of β-catenin = 0.2121 and *p*-value of total p38 = 0.1220) (Fig 3f, Figs S7–S15 in S1 File, and Tables S60–S65 in S2 File). CRE-Ter showed a non-significant trend toward inhibiting β-catenin expression, as indicated by lower average expression levels compared with Dex-treated C2C12 myotubes (*p*-values: Dex + Ter2.5 = 0.1908, Dex + Ter5 = 0.1972, Dex + Ter10 = 0.1922, and Dex + Ter20 = 0.1950). In Dex-treated C2C12 myotubes, 2.5 μg/mL CRE-Ter significantly downregulated total p38 expression (*p*-value = 0.0479). However, 5, 10, and 20 μg/mL of CRE-Ter markedly increased total p38 expression (*p*-values: Dex + Ter5 = 0.4064, Dex + Ter10 = 0.3081, and Dex + Ter20 = 0.2259). Dex and CRE-Ter had varying effect sizes on β-catenin and total p38 expression.

### Crosstalk between osteoblasts and myotubes

**Fig 4:**
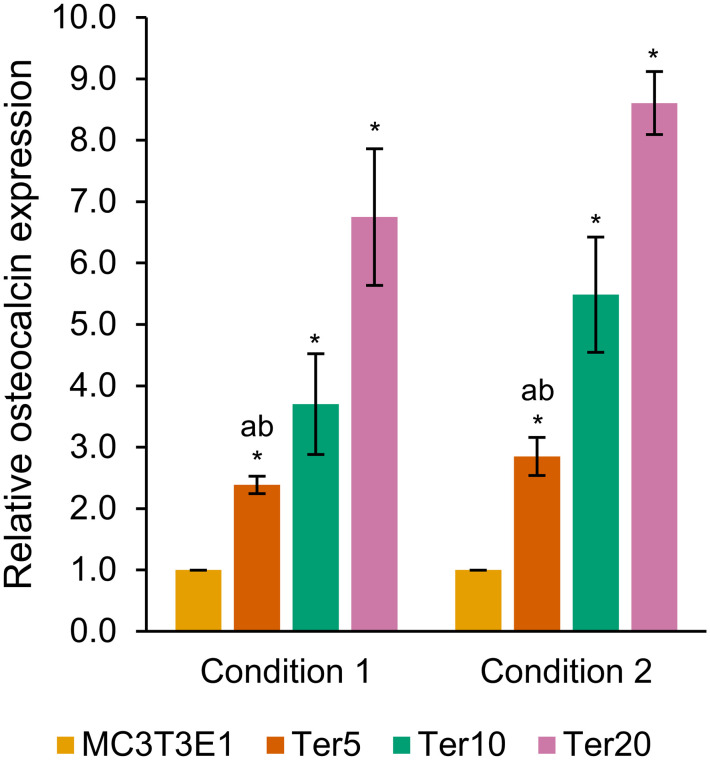
Crosstalk between osteoblasts and myotubes. MC3T3-E1 osteoblasts were cultured for 14 days under two conditions: (1) standard osteogenic differentiation medium and (2) standard osteogenic differentiation medium supplemented with 10% *v*/*v* conditioned DMEM from C2C12 myotubes. Cells were further treated with CRE-Ter at 5, 10, or 20 μg/mL (Ter5, Ter10, Ter20, respectively). Statistical significance of the difference between CRE-Ter treated and untreated MC3T3-E1 osteoblasts in the same condition is denoted as ^*^. ab: significant difference between conditions 1 and 2 at the same concentration of CRE-Ter. *p* < 0.05 and *n* = 3.

To evaluate the effect of myotube-derived factors on osteoblast differentiation, MC3T3-E1 cells were cultured under two conditions: (1) standard osteogenic differentiation medium and (2) standard osteogenic differentiation medium supplemented with 10% *v*/*v* conditioned medium from C2C12 myotubes (Fig 4 and Tables S66–S77 in S2 File). In both conditions, treatment with CRE-Ter significantly increased osteocalcin expression compared with untreated MC3T3-E1 cells. Moreover, CRE-Ter exerted a dose-dependent effect, with the highest induction observed at 20 μg/mL (*p*-values of condition 1 compared to CRE-Ter untreated cells: Ter5 = 0.0018, Ter10 = 0.0147, and Ter20 = 0.0061; *p*-values of condition 2 compared to CRE-Ter untreated cells: Ter5 = 0.0046, Ter10 = 0.0071, and Ter20 = 7.5866 × 10^−4^). MC3T3-E1 cells cultured with conditioned medium (condition 2) displayed higher osteocalcin expression than cells in standard osteogenic differentiation medium alone (condition 1), with statistical significance most evident at 5 μg/mL of CRE-Ter (*p*-values: Ter5 = 0.0336, Ter10 = 0.0644, and Ter20 = 0.0898). These results indicate that myotube-derived factors enhance osteoblast differentiation and CRE-Ter promotes osteocalcin expression in a concentration-dependent manner.

### Molecular interactions between Cu, De, and Bis with irisin

**Fig 5:**
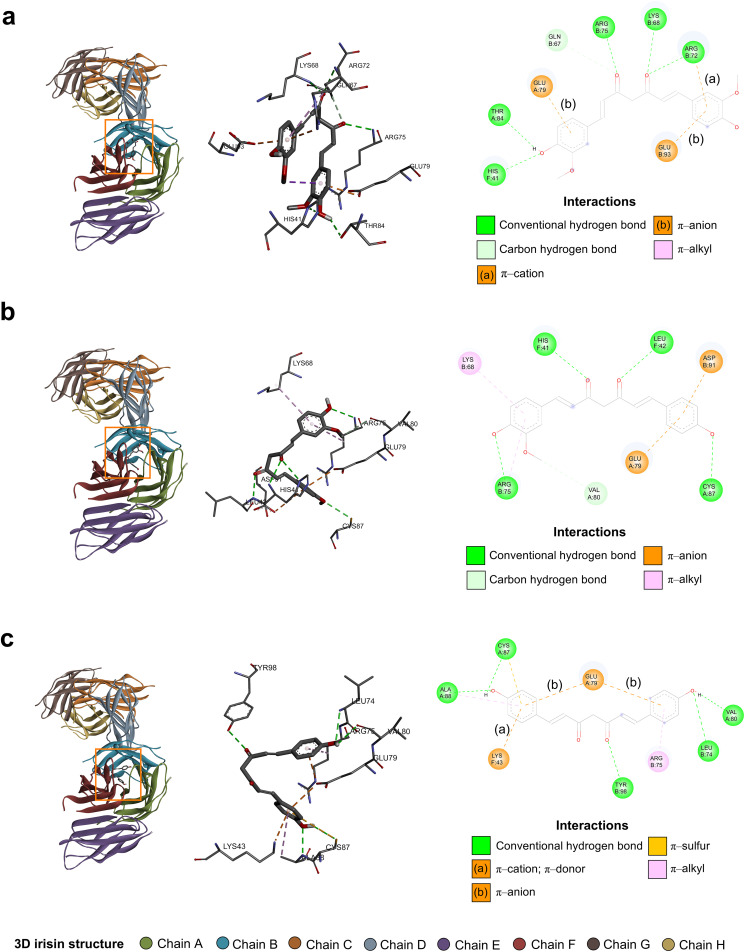
CB-Dock2 molecular docking results of irisin and curcuminoids (curcumin, demethoxycurcumin, and bisdemethoxycurcumin). **(a)** Curcumin. **(b)** Demethoxycurcumin. **(c)** Bisdemethoxycurcumin. The figures in the left panel illustrate the three-dimensional (3D) binding poses of curcuminoids. The figures in the middle panel illustrate the 3D interactions between irisin and curcuminoids. The figures in the right panel illustrate the two-dimensional (2D) diagrams of the interactions between irisin and curcuminoids. The orange rectangles denote the locations of irisin. The colored dashed lines in the 3D interactions and 2D diagrams indicate favorable intermolecular interactions between irisin and curcuminoids. The labels of the 3D interactions denote the names and positions of the contact residues. The colored circles in the 2D diagrams indicate the contact residues, along with their respective protein chains and positions. The light blue circles surrounding the colored circles in the 2D diagrams represent solvent-accessible surfaces. All figures were generated using BIOVIA Discovery Studio Visualizer.

The five binding pockets of irisin with the highest volume and their amino acid residues are shown in Table S78 and Table S79 in S2 File, respectively. The binding pockets with the greatest negative Vina scores, which indicate the strongest binding affinity, and their possible contact residues were retrieved and are presented in Fig 5 and Tables 1 and 2.

**Table 1:** Binding pockets of irisin with the most negative Vina scores after conducting molecular docking simulation with each curcuminoid molecule.

Curcuminoid	Binding pocket	Vina score (kcal/mol)	Cavity volume (Å^3^)	Docking center (x, y, z)	Docking size (x, y, z)
Curcumin	C1	−7.8	3285	−17, −42, 13	26, 26, 32
Demethoxycurcumin	C1	−8.2	3285	−17, −42, 13	27, 27, 27
Bisdemethoxycurcumin	C1	−8.0	3285	−17, −42, 13	26, 26, 32

**Table 2:** Possible contact residues of the binding pockets of irisin shown in Table 1.

Curcuminoid	Binding pocket	Possible contact residues
Curcumin	C1	Chain A: GLU79 THR82 THR84 SER86 Chain B: GLN66 GLN67 LYS68 ASP70 VAL71 ARG72 LEU74 ARG75 ASP91 GLU93 TYR98 Chain F: VAL39 ARG40 HIS41 LEU42 LYS43
Demethoxycurcumin	C1	Chain A: ILE77 GLU79 VAL80 THR82 SER86 CYS87 ALA88 TRP90 Chain B: GLN66 GLN67 LYS68 ARG72 LEU74 ARG75 TRP90 ASP91 GLU93 TYR98 Chain F: ARG40 HIS41 LEU42 LYS43
Bisdemethoxycurcumin	C1	Chain A: PHE62 ILE77 GLU79 VAL80 ASN81 THR82 THR84 SER86 CYS87 ALA88 TRP90 Chain B: GLN66 LEU74 ARG75 TRP90 ASP91 LEU92 GLU93 TYR98 Chain F: ARG40 HIS41 LEU42 LYS43

## Discussion

Our findings demonstrated that CRE-Ter promoted ALP activity, calcium deposition, and expression of Bmp-2, Runx2, and collagen 1a in MC3T3-E1 preosteoblasts significantly and dose-dependently. CRE-Ter (5, 10, and 20 µg/mL) significantly increased β-catenin expression. These results suggest that CRE-Ter promotes the differentiation of MC3T3-E1 preosteoblasts into osteoblasts through the canonical Wnt and BMP signaling pathways. CRE-Ter may trigger the interactions between Bmp-2 and BMP receptor, increasing expression of β-catenin and Runx2, secretion of ALP and collagen 1a, and deposition of calcium.

In addition to promoting osteoblast differentiation through the canonical Wnt and BMP signaling pathways, our previous studies demonstrated that CRE-Ter inhibited osteoclast differentiation by modulating the canonical NF-κB signaling pathway. Pengjam et al. reported that CRE-Ter suppressed the differentiation of RANKL-stimulated RAW264.7 macrophages through the canonical NF-κB signaling pathway, as evidenced by the inhibition of tartrate-resistant acid phosphatase, cathepsin K, p65 and IκBα phosphorylation, and the transcriptional activity of p65 [58]. Molecular docking simulations by Jantarawong et al. [59] demonstrated that Cu interacts with some contact residues of the dimerization sequences of p50, and De and Bis may interact with some contact residues of the DNA binding and dimerization sequences of p65.

NO is a pivotal messenger in bone homeostasis and skeletal muscle development. During inflammation, proinflammatory cytokines contribute to NO production through inducible NO synthase (iNOS). NO produced from osteoblasts maintains bone homeostasis. NO signaling ameliorates skeletal muscle repair and regeneration [25]. Rodríguez et al. found that in murine C2C12 myocytes, leptin triggered iNOS, leading to the promotion of the gene expression of FNDC5. Suppression of iNOS inhibited the leptin-induced increase in FNDC5 [60]. These findings suggest that iNOS mediates the cleavage of FNDC5 into irisin, thereby stimulating myogenesis. In our study, 20 µg/mL CRE-Ter prevented Dex-induced myotube atrophy, significantly downregulated TNF-α and IL-6, and increased FNDC5, irisin, and NO production in a dose-dependent manner. These results indicate that CRE-Ter alleviates cell atrophy and enhances muscle regeneration by suppressing local inflammatory responses while activating the NO–FNDC5–irisin axis. In this respect, CRE-Ter appears to mimic the beneficial effects of leptin-induced iNOS activation. However, further studies are required to fully elucidate how CRE-Ter modulates the interplay between NO signaling, FNDC5 stability, and irisin production.

Western blot analyses showed that Dex promoted the expression of β-catenin and total p38, suggesting its modulatory effect on the canonical Wnt and MAPK signaling pathways in C2C12 myotubes. CRE-Ter treatment inhibited β-catenin expression, suggesting that CRE-Ter rescues cell atrophy through the canonical Wnt signaling pathway. Nevertheless, CRE-Ter exerted a biphasic response to total p38. This may reflect the fact that the antibody used recognizes multiple isoforms (p38α, p38β, and p38γ) but not p38δ. As highlighted by Canovas and Nebreda [22], different isoforms exhibit distinct regulatory roles in myogenesis and inflammation. p38α is a critical regulator of myoblast differentiation, while p38β may play partially redundant roles in myogenesis. In addition, p38α is central in driving inflammatory cytokine production, whereas p38γ and p38δ are more associated with immune responses and tissue regeneration. Such isoform-specific functions could contribute to the heterogeneous or biphasic effects of CRE-Ter on total p38 expression in C2C12 myotubes. We acknowledge that isoform- or phosphorylation-specific analyses are needed to delineate these mechanisms more conclusively. Although measuring total p38 provides an overview of regulation in canonical MAPK signaling pathway [61–64], analyzing specific isoforms would offer deeper insights into their distinct roles in myogenesis. However, due to limited laboratory resources, we could not perform additional experiments to investigate individual p38 isoforms or their phosphorylation levels. Additionally, our data on the protein bands of phosphorylated p38 (not shown) did not appear in the blot images. Therefore, our interpretation of involvement in canonical MAPK signaling pathway should be considered preliminary, and firm conclusions about pathway activation cannot be drawn without phosphorylation-specific evidence. Further studies on the other major proteins in these signaling pathways, such as glycogen synthase kinase 3, Axin, casein kinase Iα, Dishevelled, Wnt, and adenomatous polyposis coli, are required to clarify the molecular effects of CRE-Ter on myogenesis.

Our crosstalk experiments demonstrate that factors secreted from myotubes can enhance osteoblast differentiation, as shown by the increased osteocalcin expression when MC3T3-E1 osteoblasts were cultured in a mixture of standard osteogenic differentiation medium and conditioned medium from C2C12 myotubes. This supports the concept of bidirectional communication between muscle and bone, whereby muscle-derived signals facilitate osteogenic activity. Previous studies have shown that Dex suppresses osteoblast differentiation and bone formation, contributing to osteoporosis [65,66]. In our study, although Dex was present as part of the standard osteogenic differentiation medium, CRE-Ter treatment dose-dependently restored and augmented osteocalcin expression under both culture conditions. These findings indicate that CRE-Ter not only directly promotes osteoblast differentiation but also enhances osteoblast responsiveness to myotube-derived factors despite the confounding presence of Dex. Collectively, this highlights the potential of CRE-Ter as a modulatory agent at the bone–muscle interface, contributing to the maintenance of musculoskeletal integrity in osteosarcopenia. Nevertheless, these findings remain preliminary, and deeper insight into the specific biomolecular mediators in myotube-conditioned medium, such as cytokines or myokines, is warranted.

We employed CB-Dock2 to analyze the interactions between each curcuminoid molecule and irisin. A higher negative Vina score, i.e., lower binding energy, indicates stronger interactions or more binding affinity between curcuminoid and irisin [67]. Our molecular docking results suggested that each curcuminoid molecule had a high binding affinity to some contact residues of chains A, B, and F of the binding pocket C1 of irisin, as corroborated by low Vina scores. Numerous favorable interactions, for instance, those in 2D diagrams, contributed to the highly negative binding energies of the curcuminoid–irisin interactions. Therefore, the molecular docking results support the plausibility that curcuminoids in CRE-Ter may interact with irisin at relevant residues. However, we acknowledge that docking results only predict binding affinity and do not directly demonstrate transcriptional or translational upregulation of irisin.

The FNDC5 contains two putative *N*-glycosylation sites (ASN36 and ASN81). Nie and Liu demonstrated that during C2C12 myoblast differentiation, *N*-glycosylated FNDC5 is upregulated [31,68]. Blocking *N*-glycosylation by mutating these amino acid residues reduced the stability and secretion of irisin [31,68]. Our molecular docking results showed the presence of ASN36 in binding pocket C3 of irisin and ASN81 in the other four binding pockets. Bis can interact with ASN81 of chain A of binding pocket C1. While these in silico findings suggest potential interactions at biologically relevant sites, further experimental validation is needed to determine whether such binding events directly contribute to the regulation of FNDC5 and irisin expression in Dex-treated C2C12 myotubes.

**Fig 6:**
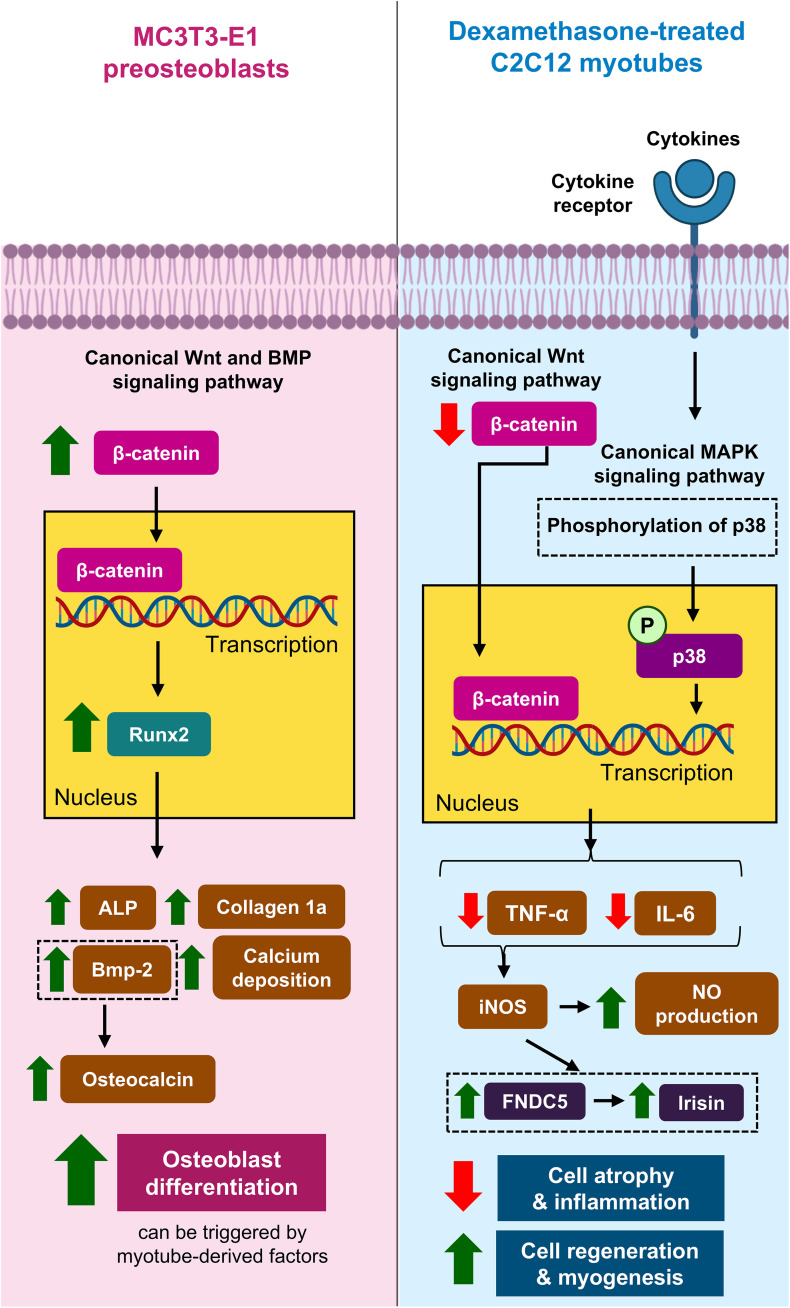
The role of CRE-Ter in the differentiation of murine bone and muscle cells. Circle labeled with “P” denotes the phosphate group. Black arrows indicate the sequences of the corresponding signaling pathway. Green up and red down arrows denote the promotion and suppression of the corresponding molecules, respectively, substantiated by this study. ALP: alkaline phosphatase. BMP: bone morphogenetic protein. FNDC5: fibronectin type III domain-containing protein 5. IL-6: interleukin-6. iNOS: inducible nitric oxide synthase. MAPK: mitogen-activated protein kinase. NO: nitric oxide. Runx2: Runt-related transcription factor 2. TNF-α: tumor necrosis factor α.

CRE-Ter increased bone and muscle cell development by promoting osteoblast differentiation and improving myotube atrophy (Fig 6), indicating its potential as a supportive herbal intervention for osteoporosis, sarcopenia, and osteosarcopenia. Because the solubility of CRE-Ter in water surpassed that of pure curcuminoids [45,46], CRE-Ter may offer advantages compared to conventional curcuminoids. However, the translational relevance of our findings requires caution. Curcumin itself is well known to suffer from poor gastrointestinal absorption, rapid metabolism, and systemic elimination, which result in very low plasma concentrations even after high oral doses in both animal and human studies [36]. Clinical trials consistently highlight the bioavailability barrier as a major limitation for therapeutic use [36]. Numerous strategies have been developed to overcome this issue, including piperine-based adjuvants, nanoparticles, liposomes, and phospholipid complexes [36]. CRE-Ter, formulated with hydroxypropyl-β-cyclodextrin and polyvinylpyrrolidone K30, represents a second-generation water-soluble system that may partially mitigate these challenges by enhancing solubility and stability [36]. Nevertheless, further research is needed to determine the pharmacokinetics, metabolic fate, and achievable therapeutic concentrations of CRE-Ter in vivo.

In addition, owing to our limited laboratory resources, further research is warranted to refine the mechanistic understanding of CRE-Ter. For example, studies on the effect of CRE-Ter and signaling inhibitors in cocultures of osteoblasts and myotubes could more accurately mimic the bone–muscle microenvironment, while investigating the crosstalk mediated by irisin between these cell types would provide additional insights. Exploring CRE-Ter’s regulation of other signaling pathways in bone and muscle cell development, along with structural characterization at the atomic level, is also essential to clarify the interactions among its major components, including curcuminoids, polyvinylpyrrolidone K30, and hydroxypropyl-β-cyclodextrin. Future studies using animal models of osteosarcopenia and eventually clinical trials will be indispensable to bridge the gap between our current in vitro findings and translational applications.

## Conclusions

Our study demonstrates that CRE-Ter, a curcuminoid-rich extract, exhibits significant improvement in bone cell differentiation, muscle cell atrophy, and biomolecular signaling. CRE-Ter effectively stimulated osteoblast differentiation in MC3T3-E1 preosteoblasts by increasing ALP activity, calcium deposition, and the expression of osteoblast markers (Bmp-2, Runx2, and collagen 1a). Additionally, CRE-Ter mitigated Dex-induced atrophy in C2C12 myotubes, improved myotube morphology, and enhanced FNDC5 and irisin expression, suggesting its role in muscle regeneration. Moreover, crosstalk experiments revealed that myotube-derived factors promote osteoblast differentiation and that CRE-Ter further augments osteocalcin expression in this context, underscoring its modulatory role at the bone–muscle interface. Molecular docking simulations further revealed strong interactions between curcuminoids and irisin, supporting CRE-Ter’s ability to modulate endogenous irisin pathways.

## Supporting information

S1 File Raw images. (PDF)

S2 File Supporting information. (PDF)
