# Supplementary material for: CRE-Ter enhances murine bone differentiation, improves muscle cell atrophy, and increases irisin expression
Source: PLoS One. 2025 Dec 4;20(12):e0338571. doi: 10.1371/journal.pone.0338571 (PMC12677536; doi:10.1371/journal.pone.0338571)
Supplement: S1 File — (PDF) [file pone.0338571.s001.pdf]

PLOS One

S1 File: Raw images

CRE-Ter enhances murine bone differentiation, improves muscle cell atrophy, and increases irisin expression

Sompot Jantarawong[1], Wipapan Khimmaktong[2], Pharkphoom Panichayupakaranant[3,4], Yutthana Pengjam[1*]

[1]Faculty of Medical Technology, Prince of Songkla University, Hat Yai, Songkhla, Thailand

[2]Division of Health and Applied Sciences, Faculty of Science, Prince of Songkla University, Hat Yai, Songkhla, Thailand

[3]Department of Pharmacognosy and Pharmaceutical Botany, Faculty of Pharmaceutical Sciences, Prince of Songkla University, Hat Yai, Songkhla, Thailand

[4]Phytomedicine and Pharmaceutical Biotechnology Excellence Center, Faculty of Pharmaceutical Sciences, Prince of Songkla University, Hat Yai, Songkhla, Thailand

[*] Corresponding author

E-mail: yutthana.p@psu.ac.th

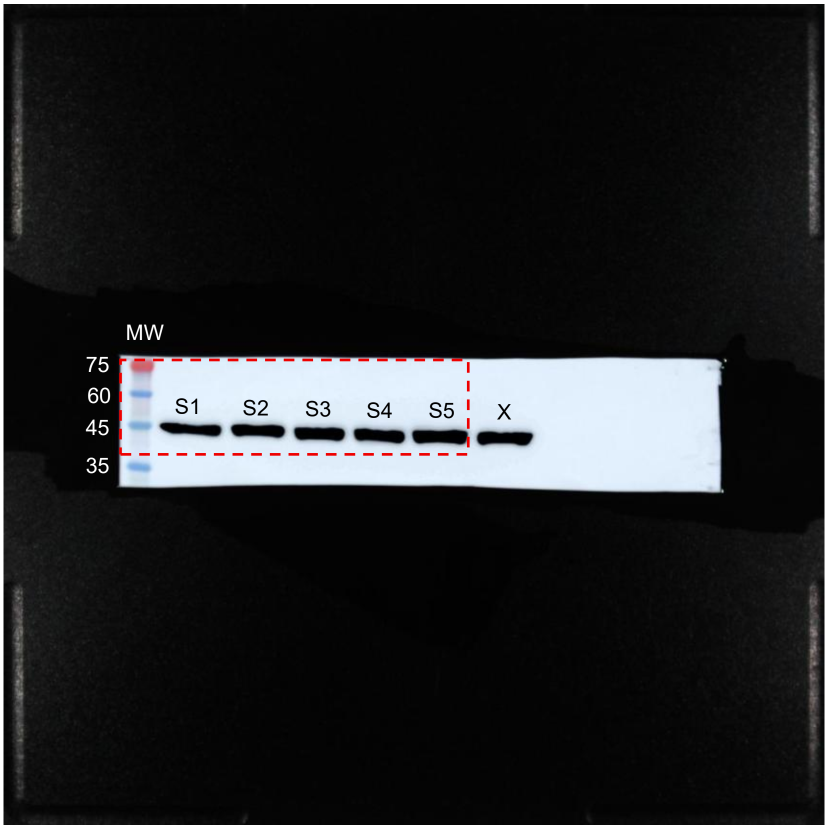

Fig S1. Original Western blot image of β-actin in MC3T3-E1 preosteoblasts (replicate 1). MW: molecular marker. S1: MC3T3-E1, S2: Ter2.5, S3: Ter5, S4: Ter10, S5: Ter20, X: unused lane. The numbers on the left of the blot indicate molecular weights of proteins in molecular marker in kilodalton (kD). The cropped blot image in the dashed rectangle is included in Fig 2e.

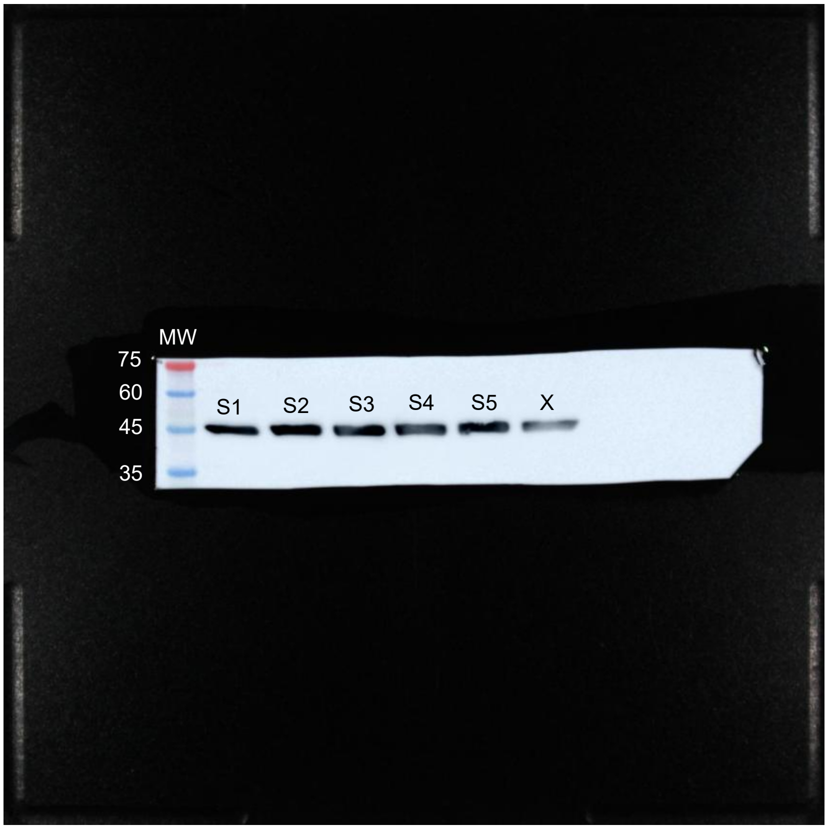

Fig S2. Original Western blot image of β-actin in MC3T3-E1 preosteoblasts (replicate 2). MW: molecular marker. S1: MC3T3-E1, S2: Ter2.5, S3: Ter5, S4: Ter10, S5: Ter20, X: unused lane. The numbers on the left of the blot indicate molecular weights of proteins in molecular marker in kilodalton (kD).

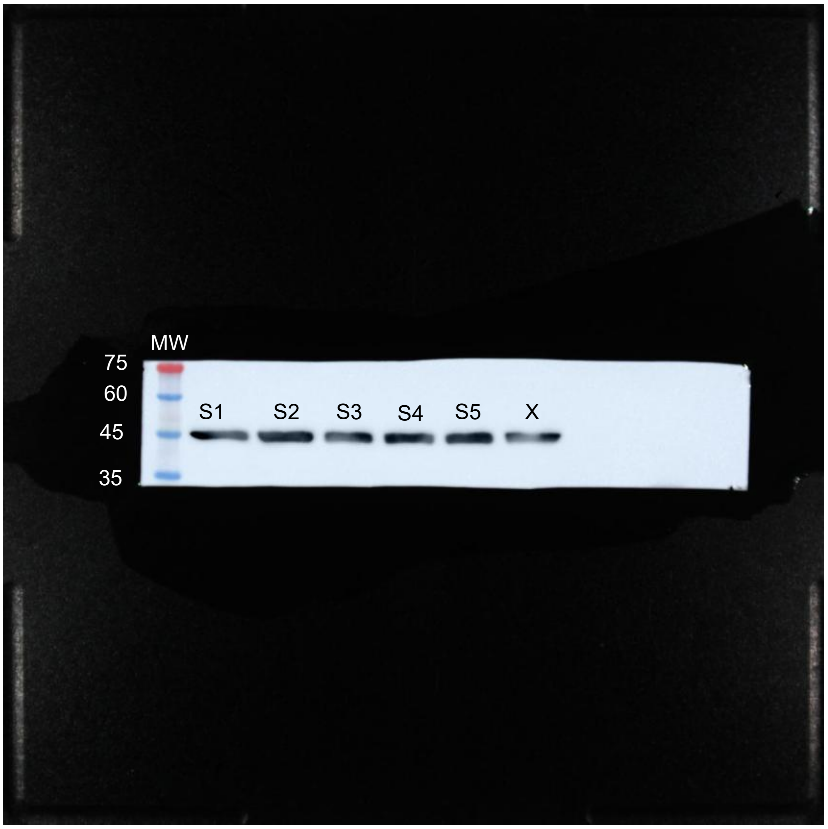

Fig S3. Original Western blot image of β-actin in MC3T3-E1 preosteoblasts (replicate 3). MW: molecular marker. S1: MC3T3-E1, S2: Ter2.5, S3: Ter5, S4: Ter10, S5: Ter20, X: unused lane. The numbers on the left of the blot indicate molecular weights of proteins in molecular marker in kilodalton (kD).

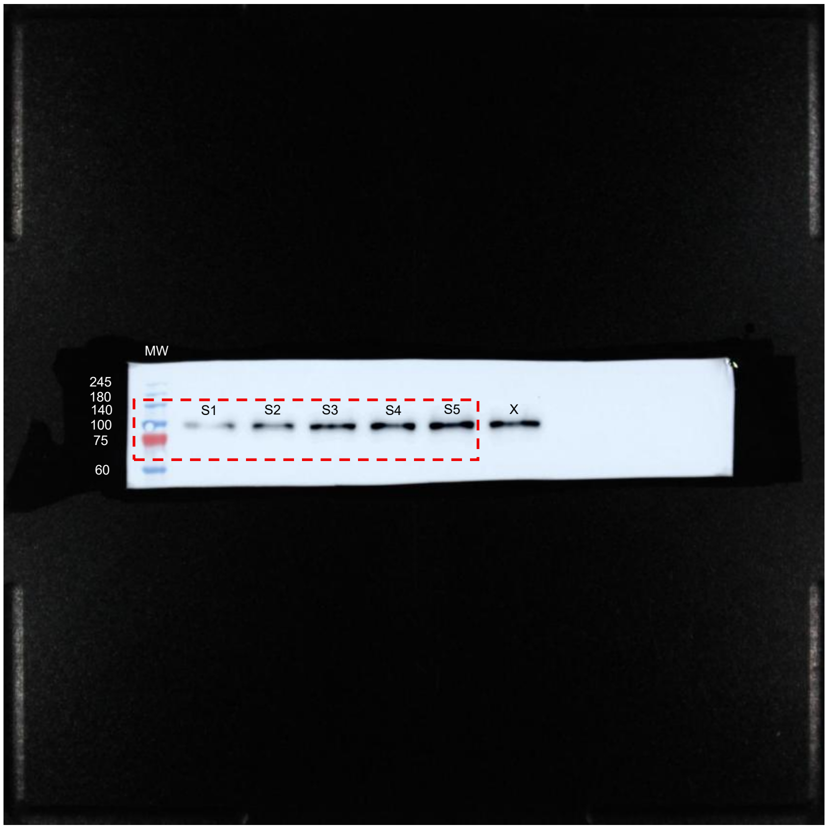

Fig S4. Original Western blot image of β-catenin in MC3T3-E1 preosteoblasts (replicate 1). MW: molecular marker. S1: MC3T3-E1, S2: Ter2.5, S3: Ter5, S4: Ter10, S5: Ter20, X: unused lane. The numbers on the left of the blot indicate molecular weights of proteins in molecular marker in kilodalton (kD). The cropped blot image in the dashed rectangle is included in Fig 2e.

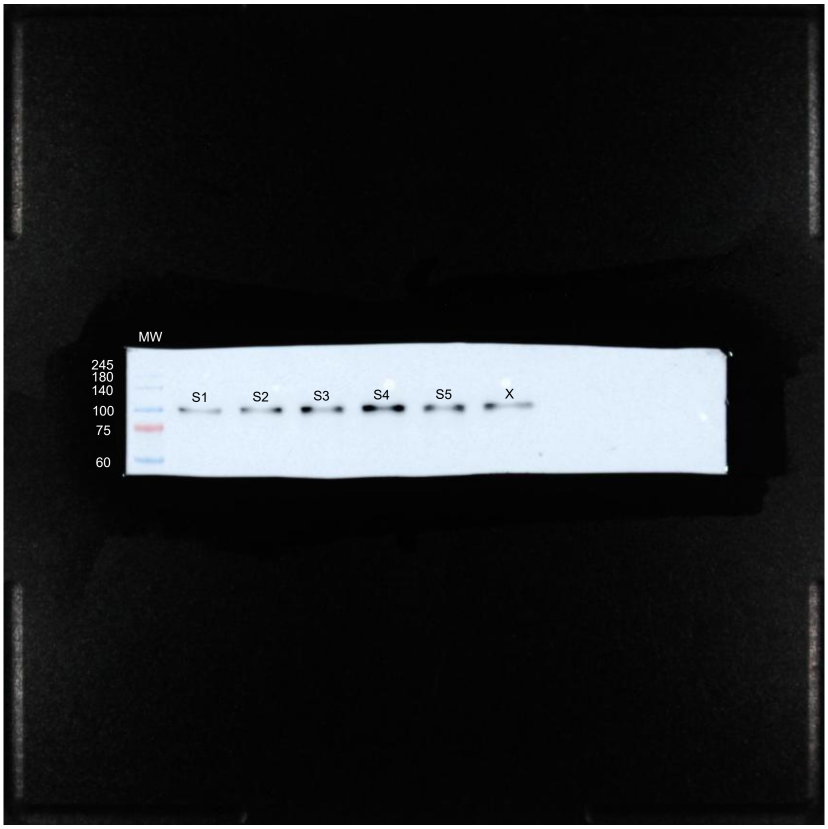

Fig S5. Original Western blot image of β-catenin in MC3T3-E1 preosteoblasts (replicate 2). MW: molecular marker. S1: MC3T3-E1, S2: Ter2.5, S3: Ter5, S4: Ter10, S5: Ter20, X: unused lane. The numbers on the left of the blot indicate molecular weights of proteins in molecular marker in kilodalton (kD).

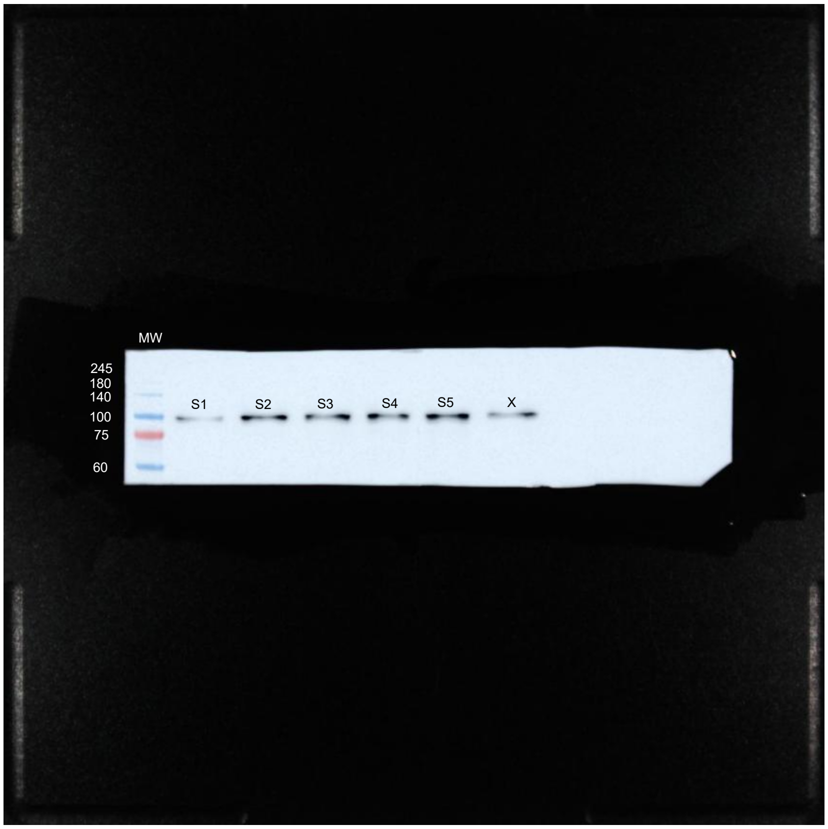

Fig S6. Original Western blot image of β-catenin in MC3T3-E1 preosteoblasts (replicate 3). MW: molecular marker. S1: MC3T3-E1, S2: Ter2.5, S3: Ter5, S4: Ter10, S5: Ter20, X: unused lane. The numbers on the left of the blot indicate molecular weights of proteins in molecular marker in kilodalton (kD).

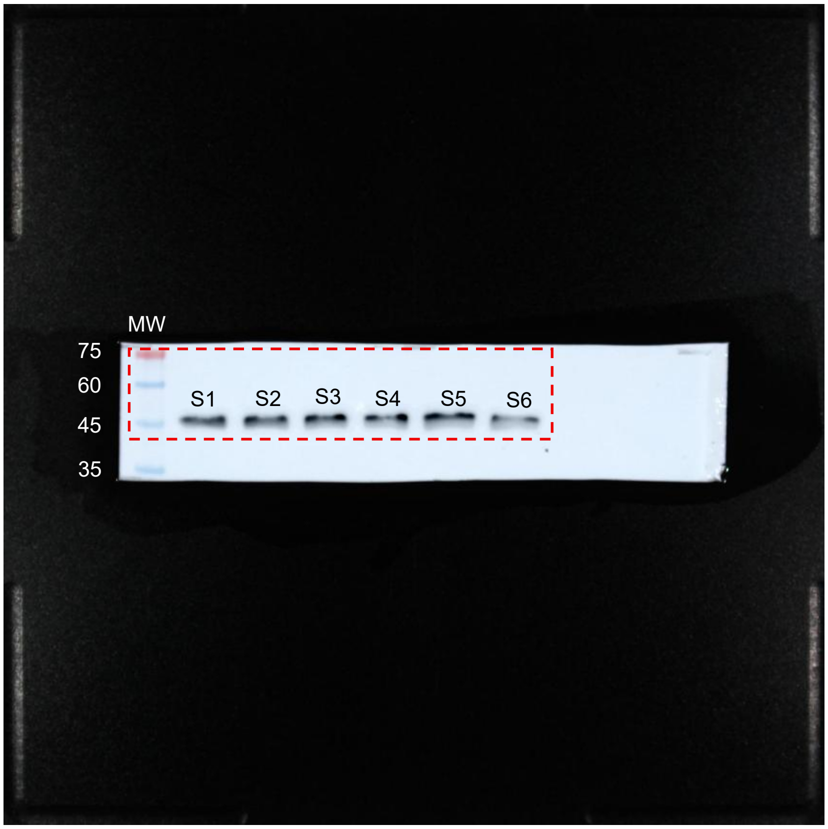

Fig S7. Original Western blot image of β-actin in C2C12 myotubes (replicate 1). MW: molecular marker. S1: C2C12 myotubes, S2: Dex, S3: Dex+Ter2.5, S4: Dex+Ter5, S5: Dex+Ter10, S6: Dex+Ter20. The numbers on the left of the blot indicate molecular weights of proteins in molecular marker in kilodalton (kD). The cropped blot image in the dashed rectangle is included in Fig 3f.

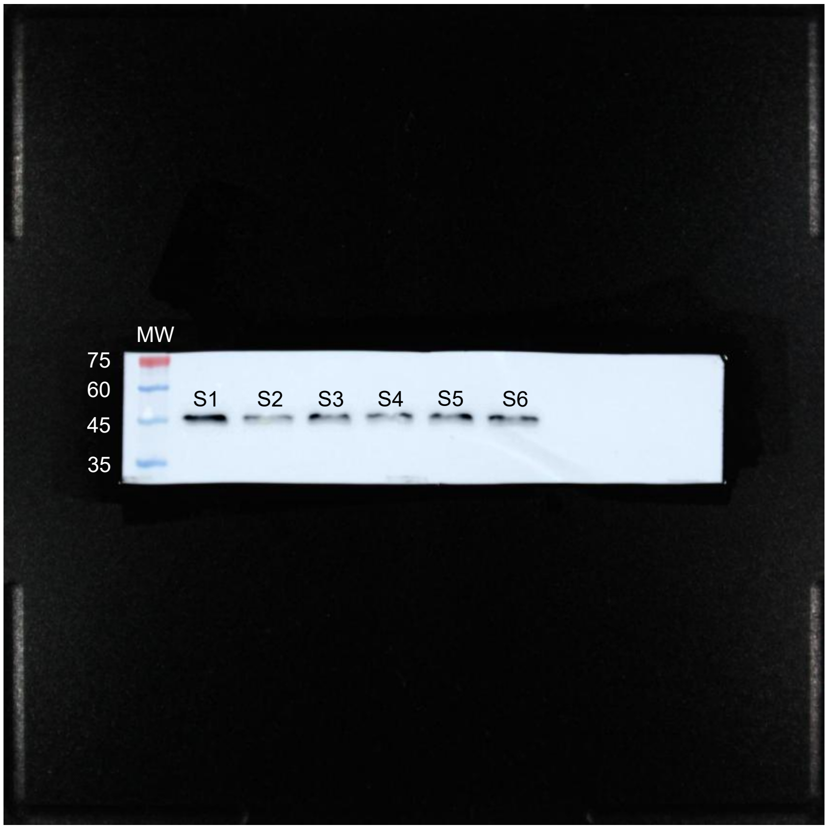

Fig S8. Original Western blot image of β-actin in C2C12 myotubes (replicate 2). MW: molecular marker. S1: C2C12 myotubes, S2: Dex, S3: Dex+Ter2.5, S4: Dex+Ter5, S5: Dex+Ter10, S6: Dex+Ter20. The numbers on the left of the blot indicate molecular weights of proteins in molecular marker in kilodalton (kD).

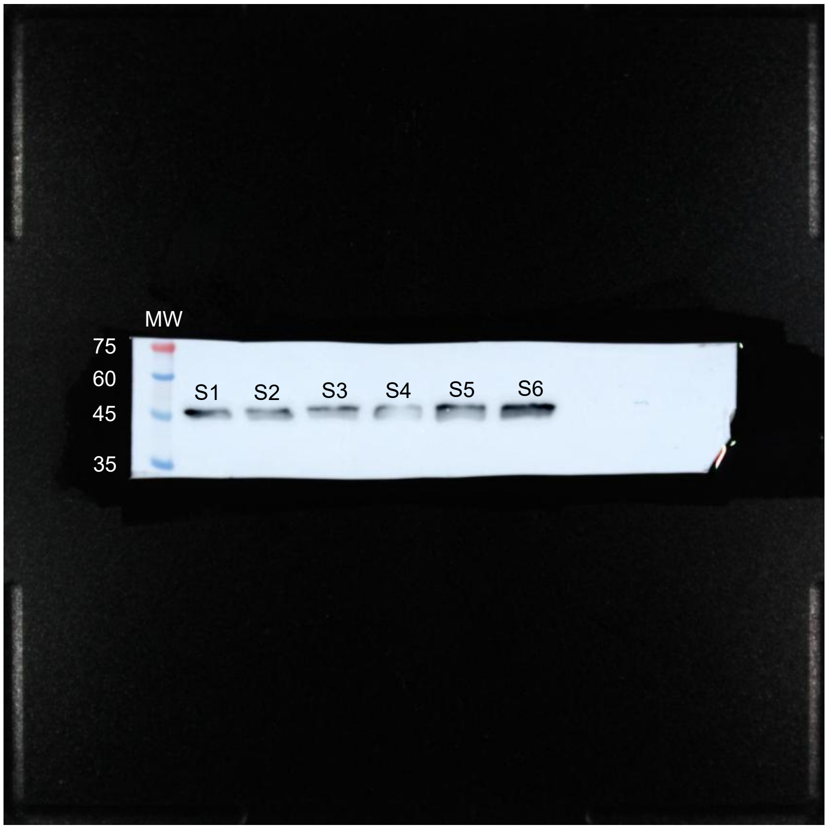

Fig S9. Original Western blot image of β-actin in C2C12 myotubes (replicate 3). MW: molecular marker. S1: C2C12 myotubes, S2: Dex, S3: Dex+Ter2.5, S4: Dex+Ter5, S5: Dex+Ter10, S6: Dex+Ter20. The numbers on the left of the blot indicate molecular weights of proteins in molecular marker in kilodalton (kD).

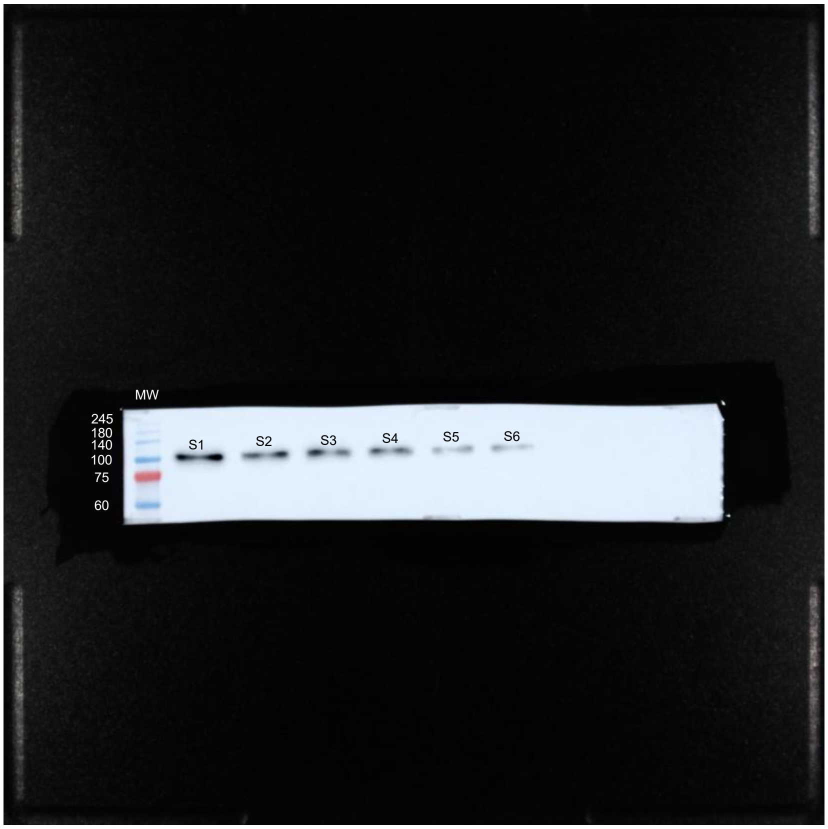

Fig S10. Original Western blot image of β-catenin in C2C12 myotubes (replicate 1). MW: molecular marker. S1: C2C12 myotubes, S2: Dex, S3: Dex+Ter2.5, S4: Dex+Ter5, S5: Dex+Ter10, S6: Dex+Ter20. The numbers on the left of the blot indicate molecular weights of proteins in molecular marker in kilodalton (kD).

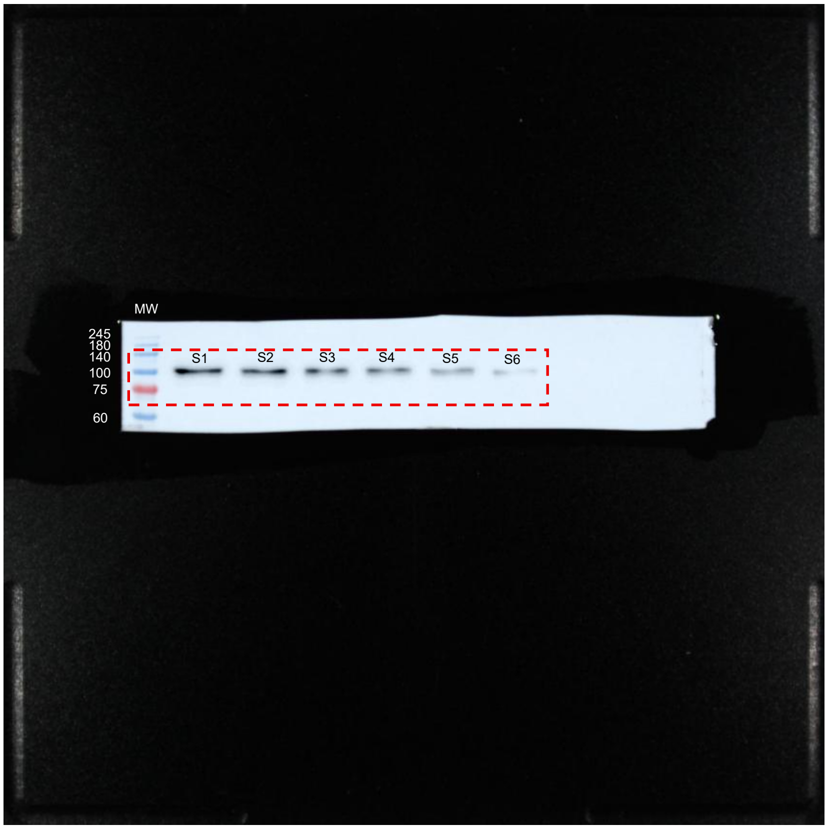

Fig S11. Original Western blot image of β-catenin in C2C12 myotubes (replicate 2). MW: molecular marker. S1: C2C12 myotubes, S2: Dex, S3: Dex+Ter2.5, S4: Dex+Ter5, S5: Dex+Ter10, S6: Dex+Ter20. The numbers on the left of the blot indicate molecular weights of proteins in molecular marker in kilodalton (kD). The cropped blot image in the dashed rectangle is included in Fig 3f.

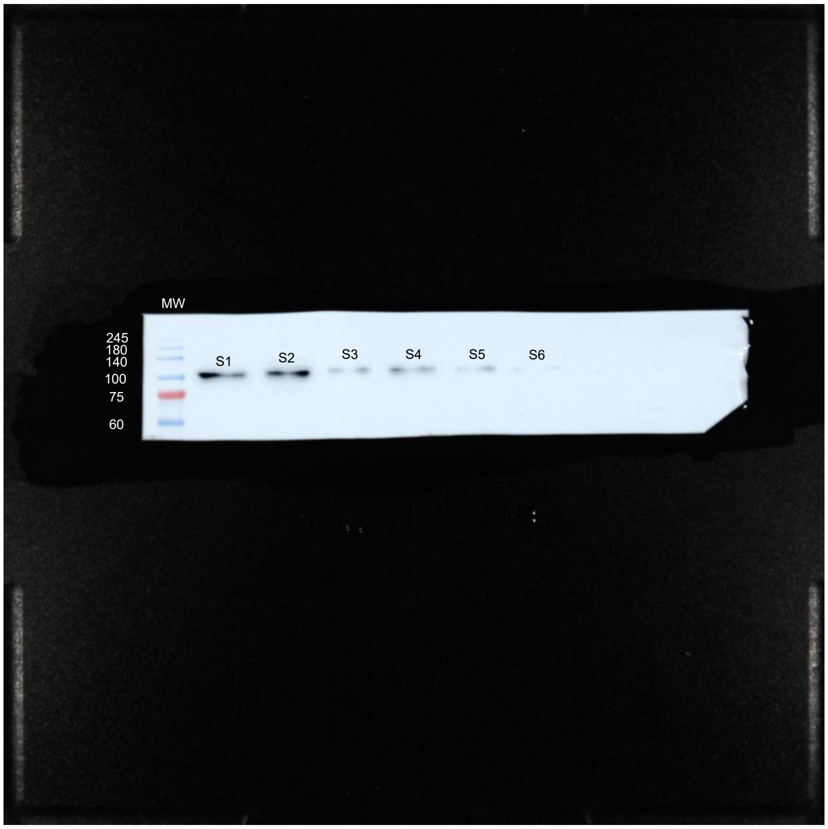

Fig S12. Original Western blot image of β-catenin in C2C12 myotubes (replicate 3). MW: molecular marker. S1: C2C12 myotubes, S2: Dex, S3: Dex+Ter2.5, S4: Dex+Ter5, S5: Dex+Ter10, S6: Dex+Ter20. The numbers on the left of the blot indicate molecular weights of proteins in molecular marker in kilodalton (kD).

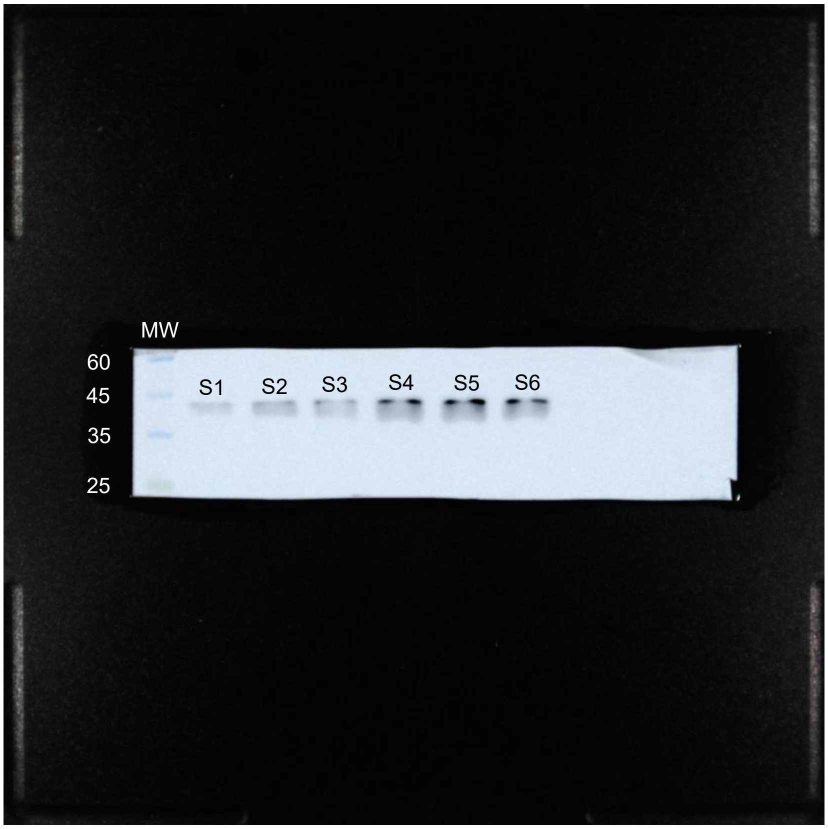

Fig S13. Original Western blot image of total p38 in C2C12 myotubes (replicate 1). MW: molecular marker. S1: C2C12 myotubes, S2: Dex, S3: Dex+Ter2.5, S4: Dex+Ter5, S5: Dex+Ter10, S6: Dex+Ter20. The numbers on the left of the blot indicate molecular weights of proteins in molecular marker in kilodalton (kD).

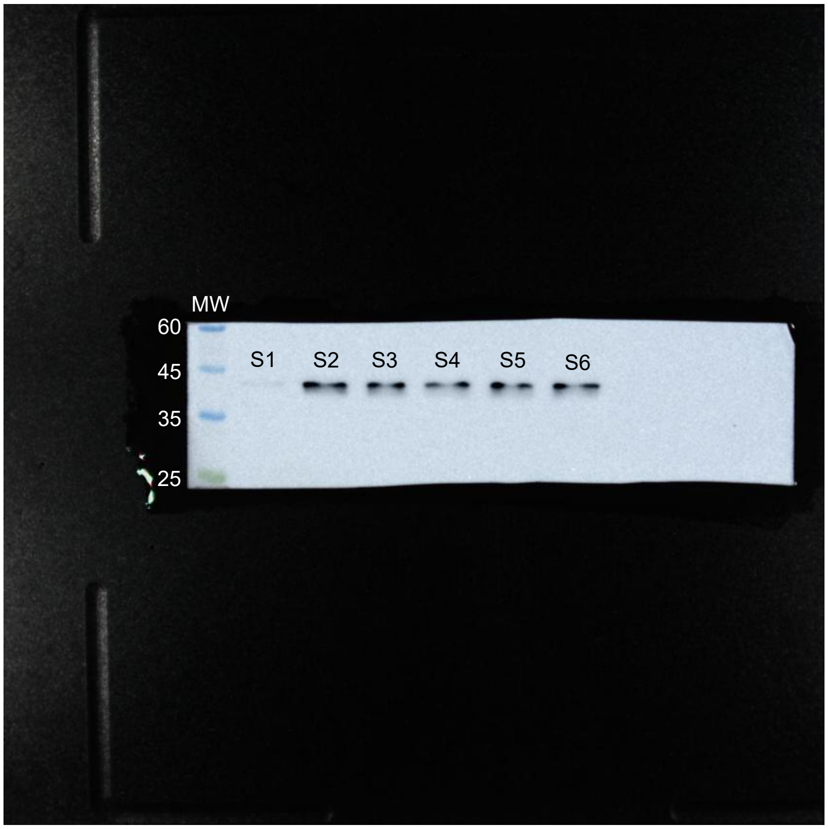

Fig S14. Original Western blot image of total p38 in C2C12 myotubes (replicate 2). MW: molecular marker. S1: C2C12 myotubes, S2: Dex, S3: Dex+Ter2.5, S4: Dex+Ter5, S5: Dex+Ter10, S6: Dex+Ter20. The numbers on the left of the blot indicate molecular weights of proteins in molecular marker in kilodalton (kD).

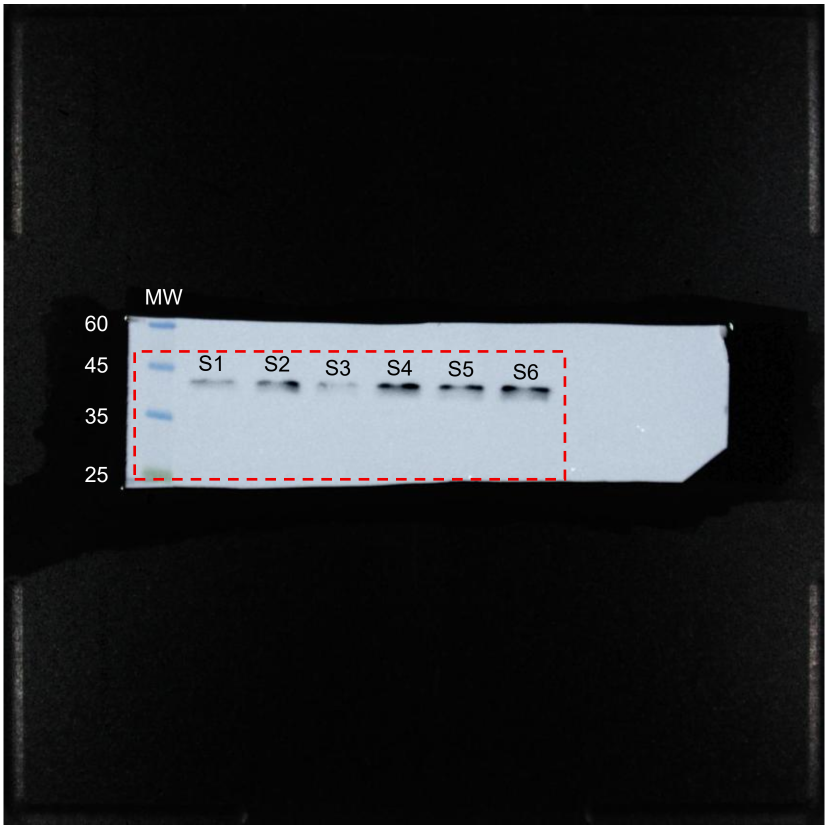

Fig S15. Original Western blot image of total p38 in C2C12 myotubes (replicate 3). MW: molecular marker. S1: C2C12 myotubes, S2: Dex, S3: Dex+Ter2.5, S4: Dex+Ter5, S5: Dex+Ter10, S6: Dex+Ter20. The numbers on the left of the blot indicate molecular weights of proteins in molecular marker in kilodalton (kD). The cropped blot image in the dashed rectangle is included in Fig 3f.
