# Supplementary material for: CRE-Ter enhances murine bone differentiation, improves muscle cell atrophy, and increases irisin expression
Source: PLoS One. 2025 Dec 4;20(12):e0338571. doi: 10.1371/journal.pone.0338571 (PMC12677536; doi:10.1371/journal.pone.0338571)
Supplement: S2 File — (PDF) [file pone.0338571.s002.pdf]

PLOS One

S2 File: Supporting information

CRE-Ter enhances murine bone differentiation, improves muscle cell atrophy, and increases irisin expression

Sompot Jantarawong[1], Wipapan Khimmaktong[2], Pharkphoom Panichayupakaranant[3,4], Yutthana Pengjam[1*]

[1]Faculty of Medical Technology, Prince of Songkla University, Hat Yai, Songkhla, Thailand

[2]Division of Health and Applied Sciences, Faculty of Science, Prince of Songkla University, Hat Yai, Songkhla, Thailand

[3]Department of Pharmacognosy and Pharmaceutical Botany, Faculty of Pharmaceutical Sciences, Prince of Songkla University, Hat Yai, Songkhla, Thailand

[4]Phytomedicine and Pharmaceutical Biotechnology Excellence Center, Faculty of Pharmaceutical Sciences, Prince of Songkla University, Hat Yai, Songkhla, Thailand

[*] Corresponding author

E-mail: yutthana.p@psu.ac.th

Table S1. Sources of cells, reagents, and instruments used

No.	Cells, reagents, or instruments	Source
**1**	**Cells**	
1.1	Murine MC3T3-E1 preosteoblasts	Biomedia Co., Ltd., Bangkok, Thailand
1.2	Murine C2C12 myoblasts	Biomedia Co., Ltd., Bangkok, Thailand
**2**	**Culture media and supplements**	
2.1	α-MEM culture medium	Gibco Co., Bangkok, Thailand
2.2	DMEM culture medium	Gibco Co., Bangkok, Thailand
2.3	Standard osteogenic differentiation medium	Sigma-Aldrich, Merck, Bangkok, Thailand
2.4	10% heat-inactivated fetal bovine serum	Gibco Co., Bangkok, Thailand
2.5	2 mM glutamine	Gibco Co., Bangkok, Thailand
2.6	100× antibiotic–antimycotic	Gibco Co., Bangkok, Thailand
2.7	2% horse serum	Gibco Co., Bangkok, Thailand
**3**	**Biochemical experiment kits**	
3.1	ALP activity kit	Primary Cell Co., Ltd., Tokyo, Japan
3.2	ALP staining kit	#AK20, Primary Cell Co Ltd., Tokyo, Japan
3.3	TRAP assay kit	TaKaRa, Bio, Inc., Tokyo, Japan
3.4	TNF-α and IL-6 ELISA kits	R&D Systems, Minneapolis, MN
3.5	RNeasy Mini Kit for RNA extraction	Qiagen, Valencia, CA, USA
3.6	ReverTra Ace qPCR Kit	Toyobo, Osaka, Japan
3.7	FastStart SYBR Green Master Mix	Roche Diagnostic, Mannheim, Germany
**4**	**Reagents for Western blotting**	
4.1	RIPA buffer	Thermo Fisher Scientific, Inc., Washington DC, USA
4.2	Antibodies	Cell Signaling Technology Inc., Beverly, MA, USA
**5**	**Instrument for CRE-Ter preparation**	
5.1	Diaion HP-20 column	Sigma-Aldrich, Merck KGaA, Darmstadt, Germany
**6**	**Spectrophotometers**	
6.1	Microplate spectrophotometer for cell viability analysis	Thermo Fisher Scientific, Multiskan FC, Pittsburgh PA, USA
6.2	Microplate spectrophotometer for detection of ALP activity	Thermo Fisher Scientific, Multiskan FC, Pittsburgh PA, USA
6.3	Spectrophotometer for absorbance detection after Alizarin red S staining	Thermo Fisher Scientific, Multiskan FC, Pittsburgh PA, USA
6.4	Microplate spectrophotometer for measurement of NO production	Infinite M200, Tecan Trading-AG, Mannedorf, Switzerland

No.	Cells, reagents, or instruments	Source
**7**	**Microscopes, digital cameras, and imaging systems**	
7.1	Brightfield microscope integrated with a digital camera for imaging after ALP staining	Model # 1X73 Olympus, Tokyo, Japan
7.2	Brightfield microscope for imaging after Alizarin red S staining	Olympus CX23, Germany
7.3	Brightfield microscope integrated with a digital camera for imaging after Wright–Giemsa staining	ZEISS, Axiocam 705 color, Thailand
7.4	EVOS M5000 inverted microscope for morphology analysis of C2C12 myotubes	Thermo Fisher Scientific, USA
7.5	Chemiluminescent image analyzer for real-time RT-PCR	Imager ChemiDoc with Image Lab 6
7.6	Chemiluminescence Vilber (Fusion Solo S) digital imaging system for Western blotting	Vilber, France
**8**	**Other reagents used in the study**	Sigma Chemical Co. and Wako Pure Chemical Industries Ltd., Bangkok, Thailand

Table S2. List of primers used for real-time RT-PCR

Gene name	Forward primer	Reverse primer
Bmp-2	5′ AGTTCTGTCCCCAGTGACGAGTTT 3′	5′ GTACAACATGGAGATTGCGCTGAGRT 3′
Runx2	5′ CCGCACGACAACCGCACCAT 3′	5′ CGCTCCGGCCCACAAATCTC 3′
Collagen 1a	5′ TTCCCTGGTGCTGATGGTGTTGCT 3′	5′ GCCTTTCCAGGTTCTCCAGCGG 3′
TNF-α	5′ GGAACACGTCGTGGGATAATG 3′	5′ GGCAGACTTTGGATGCTTCTT 3′
IL-6	5′ GGCGGATCGGATGTTGTGAT 3′	5′ GGACCCCAGACAATCGGTTG 3′
FNDC5	5′ GGCTGGGAGTTCATGTGGAA 3′	5′ TGGGAAGCGGTTATCTTTGCT 3′
Irisin	5′ ATGAAGGAGATGGGGAGGAA 3′	5′ GCGGCAGAAGAGAGCTATAACA 3′
Osteocalcin	5′ TGACAAAGCCTTCATGTCCA 3′	5′ TAGTGATACCATAGATGCGT 3′
GAPDH	5′ AAATGGTGAAGGTCGGTGTG 3′	5′ GAATTTGCCGTGAGTGGAGT 3′

Table S3. Descriptive statistics and Cohen's *d* of the MTT assay of MC3T3-E1 preosteoblasts

Condition	MC3T3-E1	Ter1	Ter2.5	Ter5	Ter10	Ter20	Ter30
Average	100.0000	100.3953	102.7559	105.3660	99.1012	101.9469	34.5931
Standard deviation	2.6471	4.4434	5.4222	9.7499	3.8291	4.0869	6.9619
Margin of error for a 95% confidence interval	2.2131	3.7148	4.5331	8.1511	3.2012	3.4168	5.8203
Lower bound of 95% confidence interval	97.7869	96.6805	98.2229	97.2149	95.9000	98.5301	28.7728
Upper bound of 95% confidence interval	102.2131	104.1101	107.2890	113.5171	102.3024	105.3636	40.4134
Cohen's *d*	Not applicable	0.1081	0.6459	0.7511	−0.2731	0.5654	−12.4190

Table S4. Shapiro-Wilk test of the MTT assay of MC3T3-E1 preosteoblasts

Condition	MC3T3-E1	Ter1	Ter2.5	Ter5	Ter10	Ter20	Ter30
p-value	0.4513	0.3178	0.2854	0.719	0.6663	0.1244	0.5399
W	0.9175	0.9007	0.8957	0.9431	0.9384	0.8579	0.9266
B	6.7085	11.1571	13.577	25.0506	9.8135	10.0151	17.7311
Skewness	0.9602	−1.0774	−0.4842	0.6826	−0.7676	−0.4486	0.5364
Skewness shape	Potentially symmetrical (p =0.202)	Potentially symmetrical (p =0.152)	Potentially symmetrical (p =0.52)	Potentially symmetrical (p =0.364)	Potentially symmetrical (p =0.307)	Potentially symmetrical (p =0.551)	Potentially symmetrical (p =0.476)
Excess kurtosis	0.5936	0.7438	−1.2433	0.6117	0.7683	−1.5984	−0.9666
Kurtosis Shape	Potentially Mesokurtic, normal like tails (p =0.689)	Potentially Mesokurtic, normal like tails (p =0.615)	Potentially Mesokurtic, normal like tails (p =0.401)	Potentially Mesokurtic, normal like tails (p =0.68)	Potentially Mesokurtic, normal like tails (p =0.604)	Potentially Mesokurtic, normal like tails (p =0.28)	Potentially Mesokurtic, normal like tails (p =0.514)
Outliers	No outliers	No outliers	No outliers	No outliers	No outliers	No outliers	No outliers

Table S5. One-way analysis of variance of the MTT assay of MC3T3-E1 preosteoblasts

Source of variation	Sum of square	Degree of freedom	Mean square	F statistic	*p*-value	F critical value
Between groups	30989.9181	6	5164.9863	156.4846	3.3543×10^{-30}	2.2904
Within groups	1617.3118	49	33.0064			
Total	32607.2299	55				

Table S6. Descriptive statistics and Cohen's *d* of ALP activity of MC3T3-E1 preosteoblasts

Condition	MC3T3-E1	Ter2.5	Ter5	Ter10	Ter20
Average	1.0063	1.2513	1.5218	1.7295	1.9747
Standard deviation	0.0870	0.0599	0.0583	0.0840	0.0431
Margin of error for a 95% confidence interval	0.0913	0.0629	0.0612	0.0881	0.0452
Lower bound of 95% confidence interval	0.9151	1.1885	1.4606	1.6414	1.9294
Upper bound of 95% confidence interval	1.0976	1.3142	1.5830	1.8176	2.0199
Cohen's *d*		3.2811	6.9620	8.4607	14.1083

Table S7. Shapiro-Wilk test of ALP activity of MC3T3-E1 preosteoblasts

Condition	MC3T3-E1	Ter2.5	Ter5	Ter10	Ter20
p-value	0.2908	0.4531	0.9947	0.2234	0.3467
W	0.8767	0.9023	0.9769	0.8619	0.8866
B	0.1821	0.1272	0.1289	0.1743	0.09078
Skewness	1.3489	−1.2763	−0.2578	−0.4995	−0.09351
Skewness shape	Potentially symmetrical (p =0.11)	Potentially symmetrical (p =0.131)	Potentially symmetrical (p =0.76)	Potentially symmetrical (p =0.555)	Potentially symmetrical (p =0.912)
Excess kurtosis	3.029	1.9666	−0.05743	−1.9298	−2.5232
Kurtosis Shape	Potentially Mesokurtic, normal like tails (p =0.082)	Potentially Mesokurtic, normal like tails (p =0.259)	Potentially Mesokurtic, normal like tails (p =0.974)	Potentially Mesokurtic, normal like tails (p =0.268)	Potentially Mesokurtic, normal like tails (p =0.147)
Outliers	1.166	No outliers	No outliers	No outliers	No outliers

Table S8. One-way analysis of variance of ALP activity of MC3T3-E1 preosteoblasts

Source of variation	Sum of square	Degree of freedom	Mean square	F statistic	*p*-value	F critical value
Between groups	3.5037	4	0.8759	186.6696	3.1507×10^{-18}	2.7587
Within groups	0.1173	25	0.0047			
Total	3.6210	29				

Table S9. Tukey's HSD test of ALP activity of MC3T3-E1 preosteoblasts

Pair	Absolute difference between the means	Standard error of the difference	Q	Lower bound of 95% confidence interval	Upper bound of 95% confidence interval	Critical mean	*p*-value
MC3T3-E1 versus Ter2.5	0.2450	0.0280	8.7609	0.1289	0.3611	0.1161	1.638×10^{-5}
MC3T3-E1 versus Ter5	0.5155	0.0280	18.4336	0.3994	0.6316	0.1161	1.337×10^{-11}
MC3T3-E1 versus Ter10	0.7232	0.0280	25.8595	0.6070	0.8393	0.1161	1.755×10^{-12}
MC3T3-E1 versus Ter20	0.9683	0.0280	34.6263	0.8522	1.0845	0.1161	1.749×10^{-12}
Ter2.5 versus Ter5	0.2705	0.0280	9.6727	0.1544	0.3866	0.1161	3.385×10^{-6}
Ter2.5 versus Ter10	0.4782	0.0280	17.0986	0.3620	0.5943	0.1161	6.115×10^{-11}
Ter2.5 versus Ter20	0.7233	0.0280	25.8655	0.6072	0.8395	0.1161	1.755×10^{-12}
Ter5 versus Ter10	0.2077	0.0280	7.4259	0.0915	0.3238	0.1161	1.755×10^{-4}
Ter5 versus Ter20	0.4528	0.0280	16.1927	0.3367	0.5690	0.1161	1.907×10^{-10}
Ter10 versus Ter20	0.2452	0.0280	8.7668	0.1290	0.3613	0.1161	1.621×10^{-5}

Table S10. Descriptive statistics and Cohen's *d* of absorbance at 550 nm of Alizarin red S staining of MC3T3-E1 preosteoblasts

Condition	MC3T3-E1	Ter2.5	Ter5	Ter10	Ter20
Average	0.0605	0.0804	0.1069	1.3505	1.5762
Standard deviation	0.0057	0.0055	0.0139	0.0648	0.0464
Margin of error for a 95% confidence interval	0.0059	0.0058	0.0146	0.0680	0.0487
Lower bound of 95% confidence interval	0.0545	0.0747	0.0923	1.2825	1.5275
Upper bound of 95% confidence interval	0.0664	0.0862	0.1215	1.4185	1.6248
Cohen's *d*		3.5800	4.3719	28.0433	45.8820

Table S11. Shapiro-Wilk test of absorbance at 550 nm of Alizarin red S staining of MC3T3-E1 preosteoblasts

Condition	MC3T3-E1	Ter2.5	Ter5	Ter10	Ter20
p-value	0.9587	0.9235	0.3611	0.3314	0.2766
W	0.964	0.9575	0.889	0.8841	0.8738
B	0.01243	0.01201	0.02934	0.1363	0.09693
Skewness	0.7053	0.03986	0.1239	−1.316	−0.4734
Skewness shape	Potentially symmetrical (p =0.404)	Potentially symmetrical (p =0.962)	Potentially symmetrical (p =0.883)	Potentially symmetrical (p =0.119)	Potentially symmetrical (p =0.575)
Excess kurtosis	1.1166	−1.4786	−2.4534	1.6683	−1.99
Kurtosis Shape	Potentially Mesokurtic, normal like tails (p =0.521)	Potentially Mesokurtic, normal like tails (p =0.396)	Potentially Mesokurtic, normal like tails (p =0.159)	Potentially Mesokurtic, normal like tails (p =0.338)	Potentially Mesokurtic, normal like tails (p =0.253)
Outliers	No outliers	No outliers	No outliers	No outliers	No outliers

Table S12. One-way analysis of variance of absorbance at 550 nm of Alizarin red S staining of MC3T3-E1 preosteoblasts

Source of variation	Sum of square	Degree of freedom	Mean square	F statistic	*p*-value	F critical value
Between groups	13.8858	4	3.4714	2627.1820	2.0958×10^{-32}	2.7587
Within groups	0.0330	25	0.0013			
Total	13.9188	29				

Table S13. Tukey's HSD test of absorbance at 550 nm of Alizarin red S staining of MC3T3-E1 preosteoblasts

Pair	Absolute difference between the means	Standard error of the difference	Q	Lower bound of 95% confidence interval	Upper bound of 95% confidence interval	Critical mean	*p*-value
MC3T3-E1 versus Ter2.5	0.0200	0.0148	1.3455	−0.0417	0.0816	0.0616	0.8738
MC3T3-E1 versus Ter5	0.0465	0.0148	3.1301	−0.0152	0.1081	0.0616	0.2076
MC3T3-E1 versus Ter10	1.2901	0.0148	86.9305	1.2284	1.3517	0.0616	1.749×10^{-12}
MC3T3-E1 versus Ter20	1.5157	0.0148	102.1372	1.4541	1.5774	0.0616	1.749×10^{-12}
Ter2.5 versus Ter5	0.0265	0.0148	1.7846	−0.0352	0.0881	0.0616	0.716
Ter2.5 versus Ter10	1.2701	0.0148	85.5851	1.2084	1.3317	0.0616	1.749×10^{-12}
Ter2.5 versus Ter20	1.4958	0.0148	100.7917	1.4341	1.5574	0.0616	1.749×10^{-12}
Ter5 versus Ter10	1.2436	0.0148	83.8005	1.1820	1.3052	0.0616	1.749×10^{-12}
Ter5 versus Ter20	1.4693	0.0148	99.0072	1.4076	1.5309	0.0616	1.749×10^{-12}
Ter10 versus Ter20	0.2257	0.0148	15.2067	0.1640	0.2873	0.0616	7.033×10^{-10}

Table S14. Descriptive statistics and Cohen's *d* of relative expression of Bmp-2 of MC3T3-E1 preosteoblasts

Condition	Ter2.5	Ter5	Ter10	Ter20
Average	1.2523	2.3773	2.5527	3.2026
Standard deviation	0.0518	0.2058	0.0683	0.0199
Margin of error for a 95% confidence interval	0.1286	0.5112	0.1697	0.0493
Lower bound of 95% confidence interval	1.1237	1.8661	2.3830	3.1532
Upper bound of 95% confidence interval	1.3810	2.8886	2.7223	3.2519
Cohen's *d*	6.8915	9.4647	32.1478	156.8362

Table S15. Shapiro-Wilk test of relative expression of Bmp-2 of MC3T3-E1 preosteoblasts

Condition	Ter2.5	Ter5	Ter10	Ter20
p-value	0.6604	0.6464	0.9864	0.1305
W	0.8954	0.8932	0.9622	0.75
B	0.0693	0.2751	0.09475	0.02432
Skewness	1.4459	−1.4564	−0.9593	1.7321
Skewness shape	Potentially symmetrical (p =0.238)	Potentially symmetrical (p =0.234)	Potentially symmetrical (p =0.433)	Potentially symmetrical (p =0.157)
Excess kurtosis	Not applicable	Not applicable	Not applicable	Not applicable
Kurtosis Shape	Not applicable	Not applicable	Not applicable	Not applicable
Outliers	No outliers	No outliers	No outliers	No outliers

Table S16. One-way analysis of variance of relative expression of Bmp-2 of MC3T3-E1 preosteoblasts

Source of variation	Sum of square	Degree of freedom	Mean square	F statistic	*p*-value	F critical value
Between groups	10.2701	4	2.5675	256.2630	5.0099×10^{-10}	3.4780
Within groups	0.1002	10	0.0100			
Total	10.3703	14				

Table S17. Tukey's HSD test of relative expression of Bmp-2 of MC3T3-E1 preosteoblasts

Pair	Absolute difference between the means	Standard error of the difference	Q	Lower bound of 95% confidence interval	Upper bound of 95% confidence interval	Critical mean	*p*-value
MC3T3-E1 versus Ter2.5	0.2523	0.0578	4.3664	−0.0166	0.5213	0.2690	0.0685
MC3T3-E1 versus Ter5	1.3773	0.0578	23.8333	1.1084	1.6463	0.2690	9.581×10^{-8}
MC3T3-E1 versus Ter10	1.5527	0.0578	26.8673	1.2837	1.8216	0.2690	2.983×10^{-8}
MC3T3-E1 versus Ter20	2.2026	0.0578	38.1132	1.9336	2.4715	0.2690	5.345×10^{-10}
Ter2.5 versus Ter5	1.1250	0.0578	19.4670	0.8560	1.3940	0.2690	6.213×10^{-7}
Ter2.5 versus Ter10	1.3003	0.0578	22.5010	1.0314	1.5693	0.2690	1.625×10^{-7}
Ter2.5 versus Ter20	1.9502	0.0578	33.7468	1.6813	2.2192	0.2690	1.972×10^{-9}
Ter5 versus Ter10	0.1753	0.0578	3.0340	−0.0936	0.4443	0.2690	0.2740
Ter5 versus Ter20	0.8252	0.0578	14.2798	0.5563	1.0942	0.2690	1.125×10^{-5}
Ter10 versus Ter20	0.6499	0.0578	11.2459	0.3809	0.9189	0.2690	9.421×10^{-5}

Table S18. Descriptive statistics and Cohen's *d* of relative expression of Runx2 of MC3T3-E1 preosteoblasts

Condition	Ter2.5	Ter5	Ter10	Ter20
Average	1.3633	2.1857	3.2403	3.9323
Standard deviation	0.0628	0.1555	0.0675	0.0845
Margin of error for a 95% confidence interval	0.1561	0.3863	0.1676	0.2099
Lower bound of 95% confidence interval	1.2073	1.7994	3.0727	3.7224
Upper bound of 95% confidence interval	1.5194	2.5720	3.4080	4.1423
Cohen's *d*	8.1794	10.7832	46.9478	49.0726

Table S19. Shapiro-Wilk test of relative expression of Runx2 of MC3T3-E1 preosteoblasts

Condition	Ter2.5	Ter5	Ter10	Ter20
p-value	0.9306	0.8716	1	1
W	0.9429	0.9305	0.9856	0.988
B	0.08627	0.2121	0.09475	0.1188
Skewness	1.1471	−1.2429	0.6104	−0.559
Skewness shape	Potentially symmetrical (p =0.349)	Potentially symmetrical (p =0.31)	Potentially symmetrical (p =0.618)	Potentially symmetrical (p =0.648)
Excess kurtosis	Not applicable	Not applicable	Not applicable	Not applicable
Kurtosis Shape	Not applicable	Not applicable	Not applicable	Not applicable
Outliers	No outliers	No outliers	No outliers	No outliers

Table S20. One-way analysis of variance of relative expression of Runx2 of MC3T3-E1 preosteoblasts

Source of variation	Sum of square	Degree of freedom	Mean square	F statistic	*p*-value	F critical value
Between groups	18.3580	4	4.5895	576.2465	8.9917×10^{-12}	3.4780
Within groups	0.0796	10	0.0080			
Total	18.4376	14				

Table S21. Tukey's HSD test of relative expression of Runx2 of MC3T3-E1 preosteoblasts

Pair	Absolute difference between the means	Standard error of the difference	Q	Lower bound of 95% confidence interval	Upper bound of 95% confidence interval	Critical mean	*p*-value
MC3T3-E1 versus Ter2.5	0.3633	0.0515	7.0516	0.1235	0.6031	0.2398	0.0039
MC3T3-E1 versus Ter5	1.1857	0.0515	23.0115	0.9459	1.4255	0.2398	1.323×10^{-7}
MC3T3-E1 versus Ter10	2.2403	0.0515	43.4806	2.0005	2.4801	0.2398	2.313×10^{-10}
MC3T3-E1 versus Ter20	2.9323	0.0515	56.9110	2.6925	3.1721	0.2398	3.811×10^{-11}
Ter2.5 versus Ter5	0.8223	0.0515	15.9599	0.5825	1.0621	0.2398	4.033×10^{-6}
Ter2.5 versus Ter10	1.8770	0.0515	36.4290	1.6372	2.1168	0.2398	8.136×10^{-10}
Ter2.5 versus Ter20	2.5690	0.0515	49.8594	2.3292	2.8088	0.2398	1.042×10^{-10}
Ter5 versus Ter10	1.0547	0.0515	20.4691	0.8149	1.2945	0.2398	3.884×10^{-7}
Ter5 versus Ter20	1.7467	0.0515	33.8994	1.5069	1.9865	0.2398	1.864×10^{-9}
Ter10 versus Ter20	0.6920	0.0515	13.4304	0.4522	0.9318	0.2398	1.961×10^{-5}

Table S22. Descriptive statistics and Cohen's *d* of relative expression of collagen 1a of MC3T3-E1 preosteoblasts

Condition	Ter2.5	Ter5	Ter10	Ter20
Average	1.1437	1.5867	2.2720	3.0467
Standard deviation	0.0215	0.0674	0.0858	0.0844
Margin of error for a 95% confidence interval	0.0534	0.1675	0.2132	0.2096
Lower bound of 95% confidence interval	1.0903	1.4192	2.0588	2.8371
Upper bound of 95% confidence interval	1.1971	1.7541	2.4852	3.2562
Cohen's *d*	9.4492	12.3062	20.9555	34.3110

Table S23. Shapiro-Wilk test of relative expression of collagen 1a of MC3T3-E1 preosteoblasts

Condition	Ter2.5	Ter5	Ter10	Ter20
p-value	1	0.8663	0.9434	0.49
W	0.9998	0.9295	0.9461	0.8659
B	0.03041	0.09192	0.1181	0.111
Skewness	−0.06974	1.2498	−1.1191	1.5625
Skewness shape	Potentially symmetrical (p =0.955)	Potentially symmetrical (p =0.308)	Potentially symmetrical (p =0.361)	Potentially symmetrical (p =0.202)
Excess kurtosis	Not applicable	Not applicable	Not applicable	Not applicable
Kurtosis Shape	Not applicable	Not applicable	Not applicable	Not applicable
Outliers	No outliers	No outliers	No outliers	No outliers

Table S24. One-way analysis of variance of relative expression of collagen 1a of MC3T3-E1 preosteoblasts

Source of variation	Sum of square	Degree of freedom	Mean square	F statistic	*p*-value	F critical value
Between groups	8.6783	4	2.1696	556.5009	1.0695×10^{-11}	3.4780
Within groups	0.0390	10	0.0039			
Total	8.7173	14				

Table S25. Tukey's HSD test of relative expression of collagen 1a of MC3T3-E1 preosteoblasts

Pair	Absolute difference between the means	Standard error of the difference	Q	Lower bound of 95% confidence interval	Upper bound of 95% confidence interval	Critical mean	*p*-value
MC3T3-E1 versus Ter2.5	0.1437	0.0361	3.9853	−0.0241	0.3114	0.1678	0.1036
MC3T3-E1 versus Ter5	0.5867	0.0361	16.2741	0.4189	0.7544	0.1678	3.362×10^{-6}
MC3T3-E1 versus Ter10	1.2720	0.0361	35.2853	1.1042	1.4398	0.1678	1.152×10^{-9}
MC3T3-E1 versus Ter20	2.0467	0.0361	56.7745	1.8789	2.2144	0.1678	3.892×10^{-11}
Ter2.5 versus Ter5	0.4430	0.0361	12.2888	0.2752	0.6108	0.1678	4.337×10^{-5}
Ter2.5 versus Ter10	1.1283	0.0361	31.2999	0.9606	1.2961	0.1678	5.12×10^{-9}
Ter2.5 versus Ter20	1.9030	0.0361	52.7892	1.7352	2.0708	0.1678	6.99×10^{-11}
Ter5 versus Ter10	0.6853	0.0361	19.0111	0.5176	0.8531	0.1678	7.768×10^{-7}
Ter5 versus Ter20	1.4600	0.0361	40.5004	1.2922	1.6278	0.1678	3.46×10^{-10}
Ter10 versus Ter20	0.7747	0.0361	21.4892	0.6069	0.9424	0.1678	2.477×10^{-7}

Table S26. Descriptive statistics and Cohen's *d* of relative expression of β-catenin of MC3T3-E1 preosteoblasts

Condition	MC3T3-E1	Ter2.5	Ter5	Ter10	Ter20
Average	1.0000	2.9860	4.0693	4.8313	4.3374
Standard deviation	0.0322	0.2110	0.1762	0.0551	0.2058
Margin of error for a 95% confidence interval	0.0799	0.5242	0.4377	0.1370	0.5113
Lower bound of 95% confidence interval	0.9201	2.4618	3.6316	4.6944	3.8261
Upper bound of 95% confidence interval	1.0799	3.5102	4.5070	4.9683	4.8487
Cohen's *d*		1.2371	2.2785	7.9796	2.1302

Table S27. Shapiro-Wilk test of relative expression of β-catenin of MC3T3-E1 preosteoblasts

Condition	MC3T3-E1	Ter2.5	Ter5	Ter10	Ter20
p-value	0.2096	0.6477	0.9568	0.3256	1
W	0.791	0.8934	0.95	0.8292	0.9932
B	0.4304	3.0002	2.5832	0.7553	3.0853
Skewness	1.7135	1.4554	1.0844	−1.6584	0.4232
Skewness shape	Potentially symmetrical (p =0.162)	Potentially symmetrical (p =0.235)	Potentially symmetrical (p =0.376)	Potentially symmetrical (p =0.176)	Potentially symmetrical (p =0.73)
Excess kurtosis	Not applicable	Not applicable	Not applicable	Not applicable	Not applicable
Kurtosis Shape	Not applicable	Not applicable	Not applicable	Not applicable	Not applicable
Outliers	No outliers	No outliers	No outliers	No outliers	No outliers

Table S28. One-way analysis of variance of relative expression of β-catenin of MC3T3-E1 preosteoblasts

Source of variation	Sum of square	Degree of freedom	Mean square	F statistic	*p*-value	F critical value
Between groups	27.8904	4	6.9726	2.5257	0.1070	3.4780
Within groups	27.6061	10	2.7606			
Total	55.4965	14				

Table S29. Descriptive statistics and Cohen's *d* of the MTT assay of Dex-treated C2C12 myotubes

Condition	C2C12 myotubes	Dex	Dex+Ter1	Dex+Ter2.5	Dex+Ter5	Dex+Ter10	Dex+Ter20	Dex+Ter30
Average	100.0000	103.1227	102.6808	102.8681	102.8220	103.5570	103.2799	36.4385
Standard deviation	1.5278	1.5644	1.7400	2.3521	2.6237	2.5262	4.2356	6.4438
Margin of error for a 95% confidence interval	1.2773	1.3079	1.4547	1.9664	2.1934	2.1119	3.5410	5.3872
Lower bound of 95% confidence interval	98.7227	101.8148	101.2261	100.9017	100.6286	101.4451	99.7389	31.0514
Upper bound of 95% confidence interval	101.2773	104.4305	104.1356	104.8344	105.0154	105.6689	106.8210	41.8257
Cohen's *d*	Not applicable	2.0195	1.6373	1.4461	1.3145	1.7039	1.0302	−13.5734

Table S30. Shapiro-Wilk test of the MTT assay of Dex-treated C2C12 myotubes

Condition	C2C12 myotubes	Dex	Dex+Ter1	Dex+Ter2.5	Dex+Ter5	Dex+Ter10	Dex+Ter20	Dex+Ter30
p-value	0.9795	0.9479	0.752	0.06058	0.9951	0.7563	0.1513	0.6644
W	0.9737	0.9672	0.946	0.8248	0.9802	0.9464	0.8667	0.9382
B	3.9888	4.0705	4.4777	5.6515	6.8725	6.502	10.4329	16.5134
Skewness	0.6364	0.2676	0.3856	1.0127	0.4289	0.4798	0.7947	0.1967
Skewness shape	Potentially symmetrical (*p* =0.397)	Potentially symmetrical (*p* =0.722)	Potentially symmetrical (*p* =0.608)	Potentially symmetrical (*p* =0.178)	Potentially symmetrical (*p* =0.568)	Potentially symmetrical (*p* =0.524)	Potentially symmetrical (*p* =0.291)	Potentially symmetrical (*p* = 0.794)
Excess kurtosis	0.6424	−1.0326	−1.156	−0.5732	0.03663	0.09145	−1.0462	−1.3567
Kurtosis Shape	Potentially Mesokurtic, normal like tails (*p* =0.664)	Potentially Mesokurtic, normal like tails (*p* =0.486)	Potentially Mesokurtic, normal like tails (*p* =0.435)	Potentially Mesokurtic, normal like tails (*p* =0.699)	Potentially Mesokurtic, normal like tails (*p* =0.98)	Potentially Mesokurtic, normal like tails (*p* =0.951)	Potentially Mesokurtic, normal like tails (*p* =0.48)	Potentially Mesokurtic, normal like tails (*p* =0.36)
Outliers	No outliers	No outliers	No outliers	No outliers	No outliers	No outliers	No outliers	No outliers

Table S31. One-way analysis of variance of the MTT assay of Dex-treated C2C12 myotubes

Source of variation	Sum of square	Degree of freedom	Mean square	F statistic	*p*-value	F critical value
Between groups	30726.9476	7	4389.5639	156.4846	$1.2399×10^{-45}$	2.2904
Within groups	602.4865	56	10.7587			
Total	31329.4342	63				

Table S32. Descriptive statistics and Cohen's *d* of TNF-α concentration of Dex-treated C2C12 myotubes

Condition	C2C12 myotubes	Dex	Dex+Ter5	Dex+Ter10	Dex+Ter20
Average	7.9250	9.9183	7.5693	4.8550	2.9563
Standard deviation	0.3066	0.6357	0.3575	0.8418	0.5149
Margin of error for a 95% confidence interval	0.7616	1.5792	0.8880	2.0912	1.2790
Lower bound of 95% confidence interval	7.1634	8.3391	6.6813	2.7638	1.6773
Upper bound of 95% confidence interval	8.6866	11.4976	8.4573	6.9462	4.2354
Cohen's *d*		3.9941	−1.0681	−4.8461	−11.7260

Table S33. Shapiro-Wilk test of TNF-α concentration of Dex-treated C2C12 myotubes

Condition	C2C12 myotubes	Dex	Dex+Ter5	Dex+Ter10	Dex+Ter20
p-value	1	0.9062	0.1626	0.447	1
W	0.9896	0.9374	0.7691	0.8574	0.9966
B	0.4313	0.8704	0.4434	1.1024	0.7269
Skewness	−0.5211	−1.1916	1.7281	−1.589	−0.3016
Skewness shape	Potentially symmetrical (p =0.67)	Potentially symmetrical (p =0.331)	Potentially symmetrical (p =0.158)	Potentially symmetrical (p =0.194)	Potentially symmetrical (p =0.805)
Excess kurtosis	Not applicable	Not applicable	Not applicable	Not applicable	Not applicable
Kurtosis Shape	Not applicable	Not applicable	Not applicable	Not applicable	Not applicable
Outliers	No outliers	No outliers	No outliers	No outliers	No outliers

Table S34. One-way analysis of variance of TNF-α concentration of Dex-treated C2C12 myotubes

Source of variation	Sum of square	Degree of freedom	Mean square	F statistic	*p*-value	F critical value
Between groups	90.0536	4	22.5134	70.3695	2.7703×10^{-7}	3.4780
Within groups	3.1993	10	0.3199			
Total	93.2529	14				

Table S35. Tukey's HSD test of TNF-α concentration of Dex-treated C2C12 myotubes

Pair	Absolute difference between the means	Standard error of the difference	Q	Lower bound of 95% confidence interval	Upper bound of 95% confidence interval	Critical mean	*p*-value
C2C12 myotubes versus Dex	1.9933	0.3266	6.1040	0.4734	3.5133	1.5199	0.0104
C2C12 myotubes versus Dex+Ter5	0.3557	0.3266	1.0891	−1.1643	1.8756	1.5199	0.9336
C2C12 myotubes versus Dex+Ter10	3.0700	0.3266	9.4009	1.5501	4.5899	1.5199	4.258×10^{-4}
C2C12 myotubes versus Dex+Ter20	4.9687	0.3266	15.2150	3.4487	6.4886	1.5199	6.283×10^{-6}
Dex versus Dex+Ter5	2.3490	0.3266	7.1931	0.8291	3.8689	1.5199	0.0034
Dex versus Dex+Ter10	5.0633	0.3266	15.5049	3.5434	6.5833	1.5199	5.276×10^{-6}
Dex versus Dex+Ter20	6.9620	0.3266	21.3190	5.4421	8.4819	1.5199	2.665×10^{-7}
Dex+Ter5 versus Dex+Ter10	2.7143	0.3266	8.3118	1.1944	4.2343	1.5199	0.0011
Dex+Ter5 versus Dex+Ter20	4.6130	0.3266	14.1259	3.0931	6.1329	1.5199	1.241×10^{-5}
Dex+Ter10 versus Dex+Ter20	1.8987	0.3266	5.8141	0.3787	3.4186	1.5199	0.0141

Table S36. Descriptive statistics and Cohen's *d* of IL-6 concentration of Dex-treated C2C12 myotubes

Condition	C2C12 myotubes	Dex	Dex+Ter5	Dex+Ter10	Dex+Ter20
Average	4.2930	6.5327	4.3257	2.2687	0.7800
Standard deviation	0.3898	0.3954	0.3905	0.3374	0.6015
Margin of error for a 95% confidence interval	0.9684	0.9822	0.9701	0.8381	1.4943
Lower bound of 95% confidence interval	3.3246	5.5505	3.3556	1.4306	−0.7143
Upper bound of 95% confidence interval	5.2614	7.5148	5.2958	3.1067	2.2743
Cohen's *d*		5.7044	0.0837	−5.5529	−6.9307

Table S37. Shapiro-Wilk test of IL-6 concentration of Dex-treated C2C12 myotubes

Condition	C2C12 myotubes	Dex	Dex+Ter5	Dex+Ter10	Dex+Ter20
p-value	1	1	0.9366	0.9911	0.6176
W	0.9982	0.9956	0.9444	0.9655	0.8884
B	0.5508	0.5579	0.5367	0.4688	0.8019
Skewness	−0.2188	−0.3432	−1.1345	−0.9205	−1.4774
Skewness shape	Potentially symmetrical (p =0.858)	Potentially symmetrical (p =0.779)	Potentially symmetrical (p =0.354)	Potentially symmetrical (p =0.452)	Potentially symmetrical (p =0.228)
Excess kurtosis	Not applicable	Not applicable	Not applicable	Not applicable	Not applicable
Kurtosis Shape	Not applicable	Not applicable	Not applicable	Not applicable	Not applicable
Outliers	No outliers	No outliers	No outliers	No outliers	No outliers

Table S38. One-way analysis of variance of IL-6 concentration of Dex-treated C2C12 myotubes

Source of variation	Sum of square	Degree of freedom	Mean square	F statistic	*p*-value	F critical value
Between groups	57.9727	4	14.4932	77.3815	1.7545×10^{-7}	3.4780
Within groups	1.8730	10	0.1873			
Total	59.8456	14				

Table S39. Tukey's HSD test of IL-6 concentration of Dex-treated C2C12 myotubes

Pair	Absolute difference between the means	Standard error of the difference	Q	Lower bound of 95% confidence interval	Upper bound of 95% confidence interval	Critical mean	*p*-value
C2C12 myotubes versus Dex	2.2397	0.2499	8.9636	1.0767	3.4026	1.1629	6.262×10^{-4}
C2C12 myotubes versus Dex+Ter5	0.0327	0.2499	0.1307	−1.1303	1.1956	1.1629	1
C2C12 myotubes versus Dex+Ter10	2.0243	0.2499	8.1018	0.8614	3.1873	1.1629	0.0014
C2C12 myotubes versus Dex+Ter20	3.5130	0.2499	14.0597	2.3501	4.6759	1.1629	1.296×10^{-5}
Dex versus Dex+Ter5	2.2070	0.2499	8.8328	1.0441	3.3699	1.1629	7.043×10^{-4}
Dex versus Dex+Ter10	4.2640	0.2499	17.0653	3.1011	5.4269	1.1629	2.154×10^{-6}
Dex versus Dex+Ter20	5.7527	0.2499	23.0232	4.5897	6.9156	1.1629	1.317×10^{-7}
Dex+Ter5 versus Dex+Ter10	2.0570	0.2499	8.2325	0.8941	3.2199	1.1629	0.0012
Dex+Ter5 versus Dex+Ter20	3.5457	0.2499	14.1904	2.3827	4.7086	1.1629	1.191×10^{-5}
Dex+Ter10 versus Dex+Ter20	1.4887	0.2499	5.9579	0.3257	2.6516	1.1629	0.0121

Table S40. Descriptive statistics and Cohen's *d* of relative expression of TNF-α of Dex-treated C2C12 myotubes

Condition	Dex	Dex+Ter5	Dex+Ter10	Dex+Ter20
Average	3.3267	2.7914	1.4508	1.0992
Standard deviation	0.1061	0.2210	0.1711	0.0105
Margin of error for a 95% confidence interval	0.2635	0.5490	0.4250	0.0261
Lower bound of 95% confidence interval	3.0632	2.2424	1.0258	1.0731
Upper bound of 95% confidence interval	3.5902	3.3405	1.8758	1.1253
Cohen's *d*	31.0190	11.4625	3.7260	13.3531

Table S41. Shapiro-Wilk test of relative expression of TNF-α of Dex-treated C2C12 myotubes

Condition	Dex	Dex+Ter5	Dex+Ter10	Dex+Ter20
p-value	1	0.9987	0.2166	0.7221
W	0.9985	0.9759	0.7939	0.9053
b	0.1499	0.3088	0.2156	0.01414
Skewness	0.1976	−0.7798	1.7107	1.3968
Skewness shape	Potentially symmetrical (p =0.872)	Potentially symmetrical (p =0.524)	Potentially symmetrical (p =0.162)	Potentially symmetrical (p =0.254)
Excess kurtosis	Not applicable	Not applicable	Not applicable	Not applicable
Kurtosis Shape	Not applicable	Not applicable	Not applicable	Not applicable
Outliers	No outliers	No outliers	No outliers	No outliers

Table S42. One-way analysis of variance of relative expression of TNF-α of Dex-treated C2C12 myotubes

Source of variation	Sum of square	Degree of freedom	Mean square	F statistic	*p*-value	F critical value
Between groups	13.4322	4	3.3580	187.6225	2.3329×10^{-9}	3.4780
Within groups	0.1790	10	0.0179			
Total	13.6111	14				

Table S43. Tukey's HSD test of relative expression of TNF-α of Dex-treated C2C12 myotubes

Pair	Absolute difference between the means	Standard error of the difference	Q	Lower bound of 95% confidence interval	Upper bound of 95% confidence interval	Critical mean	*p*-value
C2C12 myotubes versus Dex	2.3267	0.0772	30.1228	1.9672	2.6862	0.3595	8.224×10^{-9}
C2C12 myotubes versus Dex+Ter5	1.7914	0.0772	23.1932	1.4319	2.1509	0.3595	1.231×10^{-7}
C2C12 myotubes versus Dex+Ter10	0.4508	0.0772	5.8364	0.0913	0.8103	0.3595	0.0138
C2C12 myotubes versus Dex+Ter20	0.0992	0.0772	1.2847	−0.2603	0.4587	0.3595	0.8873
Dex versus Dex+Ter5	0.5352	0.0772	6.9295	0.1757	0.8947	0.3595	0.0044
Dex versus Dex+Ter10	1.8759	0.0772	24.2864	1.5164	2.2354	0.3595	8.04×10^{-8}
Dex versus Dex+Ter20	2.2274	0.0772	28.8380	1.8679	2.5869	0.3595	1.376×10^{-8}
Dex+Ter5 versus Dex+Ter10	1.3406	0.0772	17.3568	0.9811	1.7001	0.3595	1.836×10^{-6}
Dex+Ter5 versus Dex+Ter20	1.6922	0.0772	21.9085	1.3327	2.0517	0.3595	2.075×10^{-7}
Dex+Ter10 versus Dex+Ter20	0.3516	0.0772	4.5516	−0.0079	0.7111	0.3595	0.0560

Table S44. Descriptive statistics and Cohen's *d* of relative expression of IL-6 of Dex-treated C2C12 myotubes

Condition	Dex	Dex+Ter5	Dex+Ter10	Dex+Ter20
Average	3.5260	3.1030	2.0607	1.5277
Standard deviation	0.0332	0.1106	0.0977	0.0423
Margin of error for a 95% confidence interval	0.0824	0.2747	0.2426	0.1050
Lower bound of 95% confidence interval	3.4436	2.8283	1.8181	1.4227
Upper bound of 95% confidence interval	3.6084	3.3777	2.3033	1.6326
Cohen's *d*	107.7580	26.8942	15.3604	17.6610

Table S45. Shapiro-Wilk test of relative expression of IL-6 of Dex-treated C2C12 myotubes

Condition	Dex	Dex+Ter5	Dex+Ter10	Dex+Ter20
p-value	0.8781	1	0.1946	1
W	0.9317	0.9984	0.7846	0.988
b	0.04525	0.1563	0.1223	0.0594
Skewness	−1.2339	−0.203	−1.719	−0.559
Skewness shape	Potentially symmetrical (p =0.314)	Potentially symmetrical (p =0.868)	Potentially symmetrical (p =0.16)	Potentially symmetrical (p =0.648)
Excess kurtosis	Not applicable	Not applicable	Not applicable	Not applicable
Kurtosis Shape	Not applicable	Not applicable	Not applicable	Not applicable
Outliers	No outliers	No outliers	No outliers	No outliers

Table S46. One-way analysis of variance of relative expression of IL-6 of Dex-treated C2C12 myotubes

Source of variation	Sum of square	Degree of freedom	Mean square	F statistic	*p*-value	F critical value
Between groups	13.4271	4	3.3568	680.8942	3.9189×10^{-12}	3.4780
Within groups	0.0493	10	0.0049			
Total	13.4764	14				

Table S47. Tukey's HSD test of relative expression of IL-6 of Dex-treated C2C12 myotubes

Pair	Absolute difference between the means	Standard error of the difference	Q	Lower bound of 95% confidence interval	Upper bound of 95% confidence interval	Critical mean	*p*-value
C2C12 myotubes versus Dex	2.5260	0.0405	62.3122	2.3373	2.7147	0.1887	1.592×10^{-11}
C2C12 myotubes versus Dex+Ter5	2.1030	0.0405	51.8775	1.9143	2.2917	0.1887	7.937×10^{-11}
C2C12 myotubes versus Dex+Ter10	1.0607	0.0405	26.1649	0.8720	1.2493	0.1887	3.912×10^{-8}
C2C12 myotubes versus Dex+Ter20	0.5277	0.0405	13.0167	0.3390	0.7163	0.1887	2.598×10^{-5}
Dex versus Dex+Ter5	0.4230	0.0405	10.4347	0.2343	0.6117	0.1887	1.788×10^{-4}
Dex versus Dex+Ter10	1.4653	0.0405	36.1473	1.2767	1.6540	0.1887	8.823×10^{-10}
Dex versus Dex+Ter20	1.9983	0.0405	49.2955	1.8097	2.1870	0.1887	1.122×10^{-10}
Dex+Ter5 versus Dex+Ter10	1.0423	0.0405	25.7126	0.8537	1.2310	0.1887	4.653×10^{-8}
Dex+Ter5 versus Dex+Ter20	1.5753	0.0405	38.8608	1.3867	1.7640	0.1887	4.584×10^{-10}
Dex+Ter10 versus Dex+Ter20	0.5330	0.0405	13.1482	0.3443	0.7217	0.1887	2.374×10^{-5}

Table S48. Descriptive statistics and Cohen's *d* of relative expression of FNDC5 of Dex-treated C2C12 myotubes

Condition	Dex	Dex+Ter5	Dex+Ter10	Dex+Ter20
Average	0.8277	1.1660	1.5153	2.7680
Standard deviation	0.0551	0.1348	0.0913	0.1260
Margin of error for a 95% confidence interval	0.1368	0.3349	0.2268	0.3131
Lower bound of 95% confidence interval	0.6909	0.8311	1.2886	2.4549
Upper bound of 95% confidence interval	0.9645	1.5009	1.7421	3.0811
Cohen's *d*	−4.4251	1.7413	7.9840	19.8364

Table S49. Shapiro-Wilk test of relative expression of FNDC5 of Dex-treated C2C12 myotubes

Condition	Dex	Dex+Ter5	Dex+Ter10	Dex+Ter20
p-value	0.3074	0.3127	0.676	1
W	0.8242	0.8256	0.898	0.9992
b	0.0707	0.1732	0.1223	0.1782
Skewness	1.668	−1.6653	−1.4339	0.1427
Skewness shape	Potentially symmetrical (p =0.173)	Potentially symmetrical (p =0.174)	Potentially symmetrical (p =0.242)	Potentially symmetrical (p =0.907)
Excess kurtosis	Not applicable	Not applicable	Not applicable	Not applicable
Kurtosis Shape	Not applicable	Not applicable	Not applicable	Not applicable
Outliers	No outliers	No outliers	No outliers	No outliers

Table S50. One-way analysis of variance of relative expression of FNDC5 of Dex-treated C2C12 myotubes

Source of variation	Sum of square	Degree of freedom	Mean square	F statistic	*p*-value	F critical value
Between groups	7.9437	5	1.5887	203.1399	3.6607×10^{-11}	3.1059
Within groups	0.0939	12	0.0078			
Total	8.0376	17				

Table S51. Tukey's HSD test of relative expression of FNDC5 of Dex-treated C2C12 myotubes

Pair	Absolute difference between the means	Standard error of the difference	Q	Lower bound of 95% confidence interval	Upper bound of 95% confidence interval	Critical mean	*p*-value
C2C12 myotubes versus Dex	0.1723	0.0511	3.3752	−0.0702	0.4149	0.2425	0.2345
C2C12 myotubes versus Dex+Ter5	0.1660	0.0511	3.2512	−0.0765	0.4085	0.2425	0.2658
C2C12 myotubes versus Dex+Ter10	0.5153	0.0511	10.0930	0.2728	0.7579	0.2425	1.342×10^{-4}
C2C12 myotubes versus Dex+Ter20	1.7680	0.0511	34.6268	1.5255	2.0105	0.2425	1.196×10^{-10}
Dex versus Dex+Ter5	0.3383	0.0511	6.6264	0.0958	0.5809	0.2425	0.0054
Dex versus Dex+Ter10	0.6877	0.0511	13.4681	0.4451	0.9302	0.2425	7.091×10^{-6}
Dex versus Dex+Ter20	1.9403	0.0511	38.0020	1.6978	2.1829	0.2425	2.671×10^{-11}
Dex+Ter5 versus Dex+Ter10	0.3493	0.0511	6.8418	0.1068	0.5919	0.2425	0.004208
Dex+Ter5 versus Dex+Ter20	1.6020	0.0511	31.3756	1.3595	1.8445	0.2425	5.226×10^{-10}
Dex+Ter10 versus Dex+Ter20	1.2527	0.0511	24.5339	1.0101	1.4952	0.2425	9.242×10^{-9}

Table S52. Descriptive statistics and Cohen's *d* of relative expression of irisin of Dex-treated C2C12 myotubes

Condition	Dex	Dex+Ter5	Dex+Ter10	Dex+Ter20
Average	0.5113	0.7263	0.9545	1.1026
Standard deviation	0.0831	0.0306	0.0114	0.0834
Margin of error for a 95% confidence interval	0.2065	0.0759	0.0284	0.2072
Lower bound of 95% confidence interval	0.3049	0.6504	0.9261	0.8954
Upper bound of 95% confidence interval	0.7178	0.8022	0.9829	1.3097
Cohen's *d*	−8.3146	−12.6683	−5.6289	1.7394

Table S53. Shapiro-Wilk test of relative expression of irisin of Dex-treated C2C12 myotubes

Condition	Dex	Dex+Ter5	Dex+Ter10	Dex+Ter20
p-value	0.9999	0.9895	0.1305	1
W	0.9852	0.9643	0.75	0.9858
b	0.1167	0.04243	0.014	0.1171
Skewness	−0.6192	−0.9352	−1.7321	0.6071
Skewness shape	Potentially symmetrical (p =0.613)	Potentially symmetrical (p =0.445)	Potentially symmetrical (p =0.157)	Potentially symmetrical (p =0.62)
Excess kurtosis	Not applicable	Not applicable	Not applicable	Not applicable
Kurtosis Shape	Not applicable	Not applicable	Not applicable	Not applicable
Outliers	No outliers	No outliers	No outliers	No outliers

Table S54. One-way analysis of variance of relative expression of irisin of Dex-treated C2C12 myotubes

Source of variation	Sum of square	Degree of freedom	Mean square	F statistic	*p*-value	F critical value
Between groups	0.6804	4	0.1701	56.9777	7.5962×10^{-7}	3.4780
Within groups	0.0299	10	0.0030			
Total	0.7102	14				

Table S55. Tukey's HSD test of relative expression of irisin of Dex-treated C2C12 myotubes

Pair	Absolute difference between the means	Standard error of the difference	Q	Lower bound of 95% confidence interval	Upper bound of 95% confidence interval	Critical mean	*p*-value
C2C12 myotubes versus Dex	0.4887	0.0316	15.4908	0.3418	0.6355	0.1468	5.321×10^{-6}
C2C12 myotubes versus Dex+Ter5	0.2737	0.0316	8.6753	0.1268	0.4205	0.1468	8.124×10^{-4}
C2C12 myotubes versus Dex+Ter10	0.0455	0.0316	1.4424	−0.1013	0.1923	0.1468	0.8408
C2C12 myotubes versus Dex+Ter20	0.1026	0.0316	3.2514	−0.0443	0.2494	0.1468	0.2219
Dex versus Dex+Ter5	0.2150	0.0316	6.8155	0.0682	0.3618	0.1468	0.0049
Dex versus Dex+Ter10	0.4432	0.0316	14.0485	0.2963	0.5900	0.1468	1.305×10^{-5}
Dex versus Dex+Ter20	0.5912	0.0316	18.7422	0.4444	0.7381	0.1468	8.887×10^{-7}
Dex+Ter5 versus Dex+Ter10	0.2282	0.0316	7.2329	0.0813	0.3750	0.1468	0.0032
Dex+Ter5 versus Dex+Ter20	0.3762	0.0316	11.9267	0.2294	0.5231	0.1468	5.644×10^{-5}
Dex+Ter10 versus Dex+Ter20	0.1481	0.0316	4.6938	0.0012	0.2949	0.1468	0.0479

Table S56. Descriptive statistics and Cohen's *d* of nitrite concentration of Dex-treated C2C12 myotubes

Condition	C2C12 myotubes	Dex	Dex+Ter5	Dex+Ter10	Dex+Ter20
Average	0.3422	0.3449	0.2337	0.1617	0.1036
Standard deviation	0.0048	0.0087	0.0088	0.0079	0.0140
Margin of error for a 95% confidence interval	0.0051	0.0091	0.0093	0.0083	0.0147
Lower bound of 95% confidence interval	0.3371	0.3357	0.2244	0.1535	0.0890
Upper bound of 95% confidence interval	0.3472	0.3540	0.2430	0.1700	0.1183
Cohen's *d*		0.3801	−15.2520	−27.6505	−22.8142

Table S57. Shapiro-Wilk test of nitrite concentration of Dex-treated C2C12 myotubes

Condition	C2C12 myotubes	Dex	Dex+Ter5	Dex+Ter10	Dex+Ter20
p-value	0.8149	0.2277	0.3991	0.9882	0.1497
W	0.9434	0.863	0.8948	0.9729	0.8397
B	0.01047	0.01801	0.01868	0.01736	0.02865
Skewness	−0.04226	0.5488	−0.1082	0.4825	0.7486
Skewness shape	Potentially symmetrical (*p* =0.96)	Potentially symmetrical (*p* =0.516)	Potentially symmetrical (*p* =0.898)	Potentially symmetrical (*p* =0.568)	Potentially symmetrical (pval=0.376)
Excess kurtosis	−1.4886	−1.9024	−2.2198	1.0464	−1.7466
Kurtosis Shape	Potentially Mesokurtic, normal like tails (pval=0.392)	Potentially Mesokurtic, normal like tails (pval=0.274)	Potentially Mesokurtic, normal like tails (pval=0.202)	Potentially Mesokurtic, normal like tails (pval=0.548)	Potentially Mesokurtic, normal like tails (pval=0.316)
Outliers	No outliers	No outliers	No outliers	No outliers	No outliers

Table S58. One-way analysis of variance of nitrite concentration of Dex-treated C2C12 myotubes

Source of variation	Sum of square	Degree of freedom	Mean square	F statistic	*p*-value	F critical value
Between groups	0.2769	4	0.0692	798.0248	5.7298×10^{-26}	2.7587
Within groups	0.0022	25	8.6760×10^{-5}			
Total	0.2791	29				

Table S59. Tukey's HSD test of nitrite concentration of Dex-treated C2C12 myotubes

Pair	Absolute difference between the means	Standard error of the difference	Q	Lower bound of 95% confidence interval	Upper bound of 95% confidence interval	Critical mean	*p*-value
C2C12 myotubes versus Dex	0.0027	0.0038	0.7014	−0.0131	0.0185	0.0158	0.987
C2C12 myotubes versus Dex+Ter5	0.1085	0.0038	28.5285	0.0927	0.1243	0.0158	1.749×10^{-12}
C2C12 myotubes versus Dex+Ter10	0.1805	0.0038	47.4541	0.1647	0.1962	0.0158	1.749×10^{-12}
C2C12 myotubes versus Dex+Ter20	0.2386	0.0038	62.7331	0.2228	0.2543	0.0158	1.749×10^{-12}
Dex versus Dex+Ter5	0.1111	0.0038	29.2299	0.0954	0.1269	0.0158	1.749×10^{-12}
Dex versus Dex+Ter10	0.1831	0.0038	48.1555	0.1673	0.1989	0.0158	1.749×10^{-12}
Dex versus Dex+Ter20	0.2412	0.0038	63.4344	0.2254	0.2570	0.0158	1.749×10^{-12}
Dex+Ter5 versus Dex+Ter10	0.0720	0.0038	18.9256	0.0562	0.0878	0.0158	8.248×10^{-12}
Dex+Ter5 versus Dex+Ter20	0.1301	0.0038	34.2046	0.1143	0.1459	0.0158	1.749×10^{-12}
Dex+Ter10 versus Dex+Ter20	0.0581	0.0038	15.2789	0.0423	0.0739	0.0158	6.378×10^{-10}

Table S60. Descriptive statistics and Cohen's *d* of relative expression of β-catenin of Dex-treated C2C12 myotubes

Condition	C2C12 myotubes	Dex	Dex+Ter2.5	Dex+Ter5	Dex+Ter10	Dex+Ter20
Average	1.0000	2.1305	0.9717	1.0325	0.3837	0.0949
Standard deviation	0.0175	0.0650	0.0394	0.0071	0.0078	0.0172
Margin of error for a 95% confidence interval	0.0435	0.1614	0.0979	0.0177	0.0193	0.0426
Lower bound of 95% confidence interval	0.9565	1.9690	0.8737	1.0148	0.3643	0.0523
Upper bound of 95% confidence interval	1.0435	2.2919	1.0696	1.0502	0.4030	0.1375
Cohen's *d*		0.4376	-0.0190	0.0215	−0.5647	−0.9054

Table S61. Shapiro-Wilk test of relative expression of β-catenin of Dex-treated C2C12 myotubes

Condition	C2C12 myotubes	Dex	Dex+Ter2.5	Dex+Ter5	Dex+Ter10	Dex+Ter20
p-value	0.1476	0.1579	0.1671	0.1474	0.1498	0.4189
W	0.7607	0.7665	0.7715	0.7606	0.762	0.8515
B	1.7358	4.175	1.9515	0.7818	0.7833	0.1759
Skewness	1.7308	1.7291	1.7271	1.7309	1.7305	1.6058
Skewness shape	Potentially symmetrical (*p* =0.158)	Potentially symmetrical (*p* =0.158)	Potentially symmetrical (*p* =0.158)	Potentially symmetrical (*p* =0.158)	Potentially symmetrical (*p* =0.158)	Potentially symmetrical (*p* =0.19)
Excess kurtosis	Not applicable	Not applicable	Not applicable	Not applicable	Not applicable	Not applicable
Kurtosis Shape	Not applicable	Not applicable	Not applicable	Not applicable	Not applicable	Not applicable
Outliers	No outliers	No outliers	No outliers	No outliers	No outliers	No outliers

Table S62. One-way analysis of variance of relative expression of β-catenin of Dex-treated C2C12 myotubes

Source of variation	Sum of square	Degree of freedom	Mean square	F statistic	*p*-value	F critical value
Between groups	7.3619	5	1.4724	0.4695	0.7920	3.1059
Within groups	37.6325	12	3.1360			
Total	44.9945	17				

Table S63. Descriptive statistics and Cohen's *d* of relative expression of total p38 of Dex-treated C2C12 myotubes

Condition	C2C12 myotubes	Dex	Dex+Ter2.5	Dex+Ter5	Dex+Ter10	Dex+Ter20
Average	1.0000	9.6671	5.8476	10.9689	12.3005	12.7249
Standard deviation	0.0050	0.0353	0.0303	0.0052	0.0188	0.0134
Margin of error for a 95% confidence interval	0.0125	0.0876	0.0753	0.0128	0.0468	0.0332
Lower bound of 95% confidence interval	0.9875	9.5795	5.7723	10.9561	12.2537	12.6917
Upper bound of 95% confidence interval	1.0125	9.7547	5.9230	10.9817	12.3473	12.7580
Cohen's *d*		1.3506	0.8756	7.6893	3.2178	4.5632

Table S64. Shapiro-Wilk test of relative expression of total p38 of Dex-treated C2C12 myotubes

Condition	C2C12 myotubes	Dex	Dex+Ter2.5	Dex+Ter5	Dex+Ter10	Dex+Ter20
p-value	0.5869	0.87	0.1889	0.9437	0.8856	0.1856
W	0.8833	0.9302	0.782	0.9462	0.9332	0.7805
B	1.7016	12.2545	9.6603	1.8055	6.5558	4.2489
Skewness	1.4989	1.245	1.7209	−1.1183	−1.2234	1.7219
Skewness shape	Potentially symmetrical (*p* =0.221)	Potentially symmetrical (*p* =0.309)	Potentially symmetrical (*p* =0.16)	Potentially symmetrical (*p* =0.361)	Potentially symmetrical (*p* =0.318)	Potentially symmetrical (*p* =0.16)
Excess kurtosis	Not applicable	Not applicable	Not applicable	Not applicable	Not applicable	Not applicable
Kurtosis Shape	Not applicable	Not applicable	Not applicable	Not applicable	Not applicable	Not applicable
Outliers	No outliers	No outliers	No outliers	No outliers	No outliers	No outliers

Table S65. One-way analysis of variance of relative expression of total p38 of Dex-treated C2C12 myotubes

Source of variation	Sum of square	Degree of freedom	Mean square	F statistic	*p*-value	F critical value
Between groups	307.9692	5	61.5938	2.0722	0.1398	3.1059
Within groups	356.6845	12	29.7237			
Total	664.6537	17				

Table S66. Descriptive statistics and Cohen's *d* of relative expression of osteocalcin of MC3T3-E1 preosteoblasts (culture condition 1)

Sample	Ter5	Ter10	Ter20
Average	2.3867	3.7003	6.7477
Standard deviation	0.1433	0.8195	1.1129
Margin of error for a 95% confidence interval	0.3559	2.0359	2.7645
Lower bound of 95% confidence interval	2.0308	1.6645	3.9831
Upper bound of 95% confidence interval	2.7426	5.7362	9.5122
Cohen's *d*	13.6877	4.6597	7.3040

Table S67. Shapiro-Wilk test of relative expression of osteocalcin of MC3T3-E1 preosteoblasts (culture condition 1)

Sample	Ter5	Ter10	Ter20
p-value	0.9943	0.9981	1
W	0.9685	0.9744	0.9966
B	0.1994	1.1441	1.5712
Skewness	−0.8827	0.8026	0.3006
Skewness shape	Potentially symmetrical (p =0.471)	Potentially symmetrical (p =0.512)	Potentially symmetrical (p =0.806)
Excess kurtosis	Not applicable	Not applicable	Not applicable
Kurtosis Shape	Not applicable	Not applicable	Not applicable
Outliers	No outliers	No outliers	No outliers

Table S68. One-way analysis of variance of relative expression of osteocalcin of MC3T3-E1 preosteoblasts (culture condition 1)

Source of variation	Sum of square	Degree of freedom	Mean square	F statistic	*p*-value	F critical value
Between groups	54.2104	3	18.0701	37.4378	4.6809×10^{-5}	4.0662
Within groups	3.8614	8	0.4827			
Total	58.0718	11				

Table S69. Tukey's HSD test of relative expression of osteocalcin of MC3T3-E1 preosteoblasts (culture condition 1)

Pair	Absolute difference between the means	Standard error of the difference	Q	Lower bound of 95% confidence interval	Upper bound of 95% confidence interval	Critical mean	*p*-value
MC3T3-E1 versus Ter5	1.3867	0.4011	3.4571	−0.4299	3.2032	1.8166	0.1452
MC3T3-E1 versus Ter10	2.7003	0.4011	6.7321	0.8838	4.5169	1.8166	0.0062
MC3T3-E1 versus Ter20	5.7477	0.4011	14.3293	3.9311	7.5642	1.8166	3.587×10^{-5}
Ter5 versus Ter10	1.3137	0.4011	3.2751	−0.5029	3.1302	1.8166	0.1733
Ter5 versus Ter20	4.3610	0.4011	10.8723	2.5444	6.1776	1.8166	2.669×10^{-4}
Ter10 versus Ter20	3.0473	0.4011	7.5972	1.2308	4.8639	1.8166	0.0030

Table S70. Descriptive statistics and Cohen's *d* of relative expression of osteocalcin of MC3T3-E1 preosteoblasts (culture condition 2)

Sample	Ter5	Ter10	Ter20
Average	2.8493	5.4847	8.6050
Standard deviation	0.3095	0.9374	0.5137
Margin of error for a 95% confidence interval	0.7687	2.3286	1.2761
Lower bound of 95% confidence interval	2.0806	3.1561	7.3289
Upper bound of 95% confidence interval	3.6181	7.8132	9.8811
Cohen's *d*	8.4516	6.7660	20.9373

Table S71. Shapiro-Wilk test of relative expression of osteocalcin of MC3T3-E1 preosteoblasts (culture condition 2)

Sample	Ter5	Ter10	Ter20
p-value	0.994	0.617	0.1627
W	0.9682	0.8883	0.7691
B	0.4306	1.2494	0.6371
Skewness	−0.8866	1.4778	−1.7281
Skewness shape	Potentially symmetrical (p =0.469)	Potentially symmetrical (p =0.228)	Potentially symmetrical (p =0.158)
Excess kurtosis	Not applicable	Not applicable	Not applicable
Kurtosis Shape	Not applicable	Not applicable	Not applicable
Outliers	No outliers	No outliers	No outliers

Table S72. One-way analysis of variance of relative expression of osteocalcin of MC3T3-E1 preosteoblasts (culture condition 2)

Source of variation	Sum of square	Degree of freedom	Mean square	F statistic	*p*-value	F critical value
Between groups	98.3831	3	32.7944	105.9332	8.8583×10^{-7}	4.0662
Within groups	2.4766	8	0.3096			
Total	100.8597	11				

Table S73. Tukey's HSD test of relative expression of osteocalcin of MC3T3-E1 preosteoblasts (culture condition 2)

Pair	Absolute difference between the means	Standard error of the difference	Q	Lower bound of 95% confidence interval	Upper bound of 95% confidence interval	Critical mean	*p*-value
MC3T3-E1 versus Ter5	1.8493	0.3212	5.7569	0.3945	3.3041	1.4548	0.0152
MC3T3-E1 versus Ter10	4.4847	0.3212	13.9607	3.0299	5.9395	1.4548	4.353×10^{-5}
MC3T3-E1 versus Ter20	7.6050	0.3212	23.6743	6.1502	9.0598	1.4548	7.827×10^{-7}
Ter5 versus Ter10	2.6353	0.3212	8.2038	1.1805	4.0901	1.4548	0.0018
Ter5 versus Ter20	5.7557	0.3212	17.9173	4.3009	7.2105	1.4548	6.691×10^{-6}
Ter10 versus Ter20	3.1203	0.3212	9.7136	1.6655	4.5751	1.4548	5.857×10^{-4}

Table S74. One-way analysis of variance of relative expression of osteocalcin of Ter5-treated MC3T3-E1 preosteoblasts (culture conditions 1–2)

Source of variation	Sum of square	Degree of freedom	Mean square	F statistic	*p*-value	F critical value
Between groups	0.3211	1	0.3211	5.5224	0.0785	7.7086
Within groups	0.2326	4	0.0581			
Total	0.5537	5				

Table S75. One-way analysis of variance of relative expression of osteocalcin of Ter10-treated MC3T3-E1 preosteoblasts (culture conditions 1–2)

Source of variation	Sum of square	Degree of freedom	Mean square	F statistic	*p*-value	F critical value
Between groups	4.7758	1	4.7758	6.1610	0.0681	7.7086
Within groups	3.1007	4	0.7752			
Total	7.8764	5				

Table S76. One-way analysis of variance of relative expression of osteocalcin of Ter20-treated MC3T3-E1 preosteoblasts (culture conditions 1–2)

Source of variation	Sum of square	Degree of freedom	Mean square	F statistic	*p*-value	F critical value
Between groups	5.1745	1	5.1745	6.8885	0.0585	7.7086
Within groups	3.0047	4	0.7512			
Total	8.1793	5				

Table S77. Tukey's HSD test of relative expression of osteocalcin of CRE-Ter-treated MC3T3-E1 preosteoblasts (culture conditions 1–2)

Pair	Absolute difference between the means	Standard error of the difference	Q	Lower bound of 95% confidence interval	Upper bound of 95% confidence interval	Critical mean	*p*-value
Condition 1 versus condition 2 (Ter5)	0.4627	0.1392	3.3234	−0.0840	1.0093	0.5466	0.0785
Condition 1 versus condition 2 (Ter10)	1.7843	0.5083	3.5103	−0.2116	3.7803	1.9959	0.0681
Condition 1 versus condition 2 (Ter20)	1.8573	0.5004	3.7117	−0.1075	3.8221	1.9648	0.0585

Table S78. Information on irisin's five largest binding pockets

Binding pocket	Volume (Å^3)	Center (x, y, z)	Size (x, y, z)
C1	3285	−17, –42, 13	22, 26, 27
C2	1587	−11, −45, 2	26, 13, 12
C3	1352	−9, −48, 23	12, 23, 12
C4	468	−30, −9, −54	9, 1
C5	460	−12, −52, 34	10, 11, 14

Table S79. Amino acid residues of irisin's five largest binding pockets

Binding pocket	Amino acid residues
C1	Chain A: THR83 SER46 ALA47 PHE62 HSD41 ALA44 VAL80 ALA88 ASN81 THR84 VAL48 SER86 THR82 TRP90 LEU89 CYS87 ASN45 GLU79 LYS43 ASP91<br><br>Chain B: ALA44 GLU94 LEU92 LEU74 TRP90 ARG75 LYS68 ARG72 TYR98 GLN66 GLN67 ASN45 GLU93 ASP91<br><br>Chain F: ALA44 HSD41 PHE119 VAL39 GLU126 ARG123 GLU124 THR121 ARG40 PRO122 ALA125 LYS127 LYS120 ASN45 ASP95 LYS43 LEU42
C2	Chain A: LEU92 LEU74 TRP90 ARG75 LYS68 ARG72 TYR98 GLN66 GLN67 ASP91<br><br>Chain B: CYS87 ALA47 PHE62 HSD41 THR84 VAL80 ALA88 VAL48 SER86 THR82 TRP90 ASN81 SER46 ASN45 GLU79 LYS43<br><br>Chain F: GLU126 ARG123 GLU124 LYS127
C3	Chain A: SER50 THR83 TRP51 THR84 ASP52 SER86 ARG40 ASN36 VAL39 THR38 ARG85<br><br>Chain E: SER46 LEU92 TRP90 ASN45 ASP91 LYS43 GLU93<br><br>Chain F: SER50 ALA47 HSD41 ALA88 VAL48 ARG40 SER86 TRP90
C4	Chain G: CYS87 PHE62 THR84 ALA88 SER86 THR82 VAL80 ASN81 GLU79<br><br>Chain H: LEU92 LEU74 TRP90 ARG75 LYS68 ARG72 TYR98 GLN66 GLN67 GLU93 VAL71 ASP91
C5	Chain E: LEU92 LEU74 TRP90 ARG75 LYS68 ARG72 TYR98 GLN66 GLN67 GLU93 VAL71 ASP91<br><br>Chain F: CYS87 PHE62 THR84 VAL80 ALA88 SER86 THR82 TRP90 ASN81 ILE77 GLU79
